# Variable A-type lamin expression in Merkel cell carcinoma cell lines and its association with nuclear integrity

**DOI:** 10.1038/s41598-026-39775-y

**Published:** 2026-02-25

**Authors:** Merel Stiekema, Charlotte van Gorp, Amanda Macamo, Axel zur Hausen, Marc A. M. J. van Zandvoort, Frans C. S. Ramaekers, Jos L. V. Broers

**Affiliations:** 1 Department of Genetics and Cell Biology, GROW-Research Institute for Oncology and Reproduction, Maastricht University Medical Centre, Maastricht, The Netherlands; 2 Department of Pathology, GROW-Research Institute for Oncology and Reproduction, Maastricht University Medical Center, Maastricht, The Netherlands; 3 Institute for Molecular Cardiovascular Research IMCAR, RWTH Aachen University, Aachen, Germany; 4 CARIM-Cardiovascular Research Institute Maastricht, Maastricht University Medical Centre, Maastricht, The Netherlands

**Keywords:** Merkel cell carcinoma, Polyoma virus, Nuclear lamins, Nuclear rupture, Cancer, Cell biology, Molecular biology, Oncology

## Abstract

Merkel cell carcinoma (MCC) is an aggressive type of neuro-endocrine skin cancer and can be subdivided into Merkel cell polyomavirus (MCPyV)-positive and MCPyV-negative cases. The nuclear lamina, consisting of A- and B-type lamins, is altered in different types of cancer and can be disrupted by viral infections. This study investigated A- and B-type lamin expression levels in MCPyV-negative and MCPyV-positive MCC cell lines in relation to the MCPyV status, using (live cell) imaging, qPCR, and Western blotting. MCPyV-positive MCC (non-adherent) cells showed lower A-type lamin and higher B-type lamin expression at both protein and mRNA levels as compared to MCPyV-negative (adherent) cells. Nuclear envelope (NE) ruptures were reversible in MCPyV-negative cells, while in MCPyV-positive cells these lead to cell death. Transfection of MCPyV-negative cells with small (sT) or large tumor (LT) antigens did not result in the expected decrease of A-type lamin expression. Future research into the correlation between A-type lamin expression levels and the different growth patterns, the occurrence of NE ruptures, the (lack of) NE repair in MCC cell lines, and the relation of lamin expression and tumor metastasis, will contribute to a better understanding of the molecular background of the aggressive phenotype of MCC.

**Supplementary Information:**

The online version contains supplementary material available at 10.1038/s41598-026-39775-y.

## Introduction

Merkel cell carcinoma (MCC) is an aggressive type of skin cancer with a high metastatic potential and poor prognosis^1^. Although MCC is considered uncommon, its incidence has increased drastically in the past years and is expected to rise even further in the coming years^2,3^. This increase is partly related to advanced age, which is one of the risk factors for MCC, with the highest prevalence among adults aged 75 years and older^4^. Other risk factors for MCC include chronic UV exposure, immunosuppression, and infection with the Merkel cell polyomavirus (MCPyV)^1,5^. In the Northern hemisphere, MCPyV is found to be integrated in approximately 80% of MCC^1,6^. In contrast, in Australia this is as low as 18–24%^7,8^, while this country has the highest MCC incidence rate, probably due to a high UV index^4^. MCC can be subdivided into two molecular subclasses, i.e. MCPyV-positive MCC and MCPyV-negative MCC^3^. Patients with MCPyV-negative MCC tumors have a worse prognosis as compared to those with MCPyV-positive tumors, due to the high aggressiveness and higher frequency of nodal or distant metastasis^9,10^.

MCPyV is a non-enveloped, double-stranded DNA virus belonging to the family of *Polyomaviridae*^11^. It is considered part of the human skin virome and seropositivity is very common in the population^12^. The double-stranded, circular genome of MCPyV consists of two transcriptional units, the early and late regions^13^. The genes of the early region are transcribed immediately after infection and yield four spliced mRNAs encoding four proteins, including the small and large tumor antigens (sT and LT)^13,14^. These proteins are involved in the replication of the viral genome, including the genes of the late region that encode for capsid proteins VP1 and VP2, which are required for the assembly of new virions^14,15^.

For MCPyV-mediated carcinogenesis of MCC, integration of the viral genome into the host DNA, as well as the expression of a truncated form of LT, is required^13,14,16,17^. As there is a low probability of both events occurring simultaneously, this might explain the low incidence of MCC as compared to the high prevalence of MCPyV seropositivity^17^. The mutation of LT is causing a truncation of the full-length LT protein, specifically a loss of the original LT C-terminus^14^. Truncated LT prevents viral replication and facilitates cell proliferation, by amongst others permanently inactivating the Rb protein^14–16^. After clonal integration of MCPyV, sT remains to be expressed, which is also found to contribute to oncogenesis in several ways (see for review e.g^15^. For example, sT inhibits the tumor suppressor 4E-BP1, which is important for cell transformation^18^. MCPyV-negative MCCs show highly recurrent inactivation of tumor suppressor genes, including *TP53*, *RB1*, and genes encoding members of the Notch family of signaling proteins^13,17^.

Lamins have been found to play an important role in tumor cell invasion and metastasis^19–25^. Furthermore, for several viruses it has been demonstrated that the nuclear lamina is altered upon virus entry and/or egress of the nucleus (reviewed in^26^. In mouse polyomavirus infections, A- and B-type lamins have been described to contribute to both viral replication and nuclear egress^27^. A- and B-type nuclear lamins are part of the nuclear lamina, together with the lamin-associated proteins, and are located underneath the inner nuclear membrane. The major isoforms of A-type lamins are lamin A and lamin C, splice variants encoded by the same *LMNA* gene, while for B-type lamins these are lamin B1 and B2, encoded by the *LMNB1* and *LMNB2* gene, respectively^28–30^. Lamins interact with a plethora of proteins and are involved in many cellular processes and functions (see for reviews^26,31–33^. Mutations in the genes that code for lamin proteins can lead to a group of rare diseases with distinct phenotypes, collectively called laminopathies^34,35^. Fluorescence staining of lamins in cells obtained from laminopathy patients reveals abnormalities in lamin localization, visible as nuclear blebs, honeycomb-like structures, micronuclei, and donut-shaped nuclei^36,37^. Abnormalities in nuclear morphology are also a hallmark in cancer and may occur due to several mechanisms, including aneuploidy, alterations in gene expression or nucleo-cytoplasmic transport, increased cytoskeletal stress, histone modifications, and alterations in nuclear lamin levels^38–41^. Differential lamin expression has been reported in a variety of cancers. Both increased^42–44^, decreased^45–51^, and variable^49,52,53^ levels of A-type lamins have been reported, depending on the type of cancer. Similarly, for lamin B1^54–57^ and lamin B2^58–60^ upregulated levels have been reported in several tumor types. For lamin B1, also lower expression levels have been reported in malignancies of the lung, breast, and gastrointestinal tract^24,48,49,51,61^. Skin cancers other than MCC have been studied for their A- and B-type lamin expression. Elevated A-type lamin levels were found in the basal cell layer of basal cell carcinoma (BCC) and squamous cell carcinoma (SCC)^62^, while a low level of A-type lamins was reported in the highly proliferative cells of BCC^63^. Furthermore, MCC is phenotypically similar to SCLC, which has been demonstrated to exhibit low A-type lamin^51^ and lamin B1 expression^24^, as well as irreversible nuclear envelope (NE) ruptures that lead to cell death^26^. If regions of the NE are more fragile, for instance due to mutated lamins or altered lamin levels, higher NE rupture rates are found in these cells^64,65^. In oropharyngeal tumors, that present as HPV-positive or HPV-negative, lamin B1 expression appeared to be differentially expressed between these types^66^.

The differential A-and B-type lamin expression is hypothesized to contribute to cancer progression in several ways. As mentioned above, lamins are involved in metastasis, where migration of cancer cells can be facilitated by a low expression of A-type lamins, as this makes the nucleus more flexible^67,68^. Increased lamin levels on the other hand are important for mechanical stability^69–71^, which could restrain mechanical stress in solid tumors or in the bloodstream during metastasis^72^. Alternatively, it could upregulate actin-bundling protein T-plastin and downregulate cell adhesion molecule E-cadherin to increase cell motility, as reported for colorectal cancer^73^. A study by Wang et al.^74^ points towards the importance of a correctly balanced level of A-type lamin expression, as shown in an ovarian cancer cell line that demonstrated the highest migration rate when lamin A/C expression was inhibited by 30–40%, but a decreased migration at 70–80% inhibition or lamin A/C overexpression. Since its expression differs greatly between tumors it is not feasible to employ a decreased or increased lamin expression pattern as a diagnostic or prognostic biomarker for cancer in general.

To assess the effects of these lamin expression patterns in MCC and its potential relation to MCPyV, this study aimed to investigate the A- and B-type lamin expression in MCPyV-positive and -negative MCC cell lines. Specifically, A- and B-type lamin expression was determined for MCPyV-positive (MKL-1 and MKL-2) and MCPyV-negative (MCC13 and MCC26) cell lines using immunofluorescence, Western blotting, and Reverse Transcriptase quantitative Polymerase Chain Reaction (RT-qPCR). Immunofluorescence staining of lamins and emerin, in combination with Confocal Laser Scanning Microscopy (CLSM), allowed for detailed investigation of not only the lamin expression levels, but also the study of lamin localization and nuclear morphology. Additionally, NE ruptures were investigated using live cell imaging and by determining the relocalization of PML Nuclear Bodies to the cytoplasm. Finally, the effect of MCPyV on lamin expression was further assessed by transfecting a MCPyV-negative cell line with constructs encoding the viral oncogenes sT and LT.

## Results

### Differential A- and B-type lamin expression in MCPyV-positive and MCPyV-negative MCC cell lines

#### Immunofluorescence staining

The MCPyV status of the MCPyV-negative and -positive cell lines could be confirmed by absence of staining in MCC13 and MCC26 and positive MCPyV-LT antigen immunofluorescence reactivity in MKL-1 and MKL-2, respectively (Supplemental Figure S1). Additionally, while the MCPyV-positive cells demonstrated clear staining for the MCC marker cytokeratin 20 (CK20), MCPyV-negative cells were negative for CK20 (Supplemental Figure S2).

**Fig. 1:**
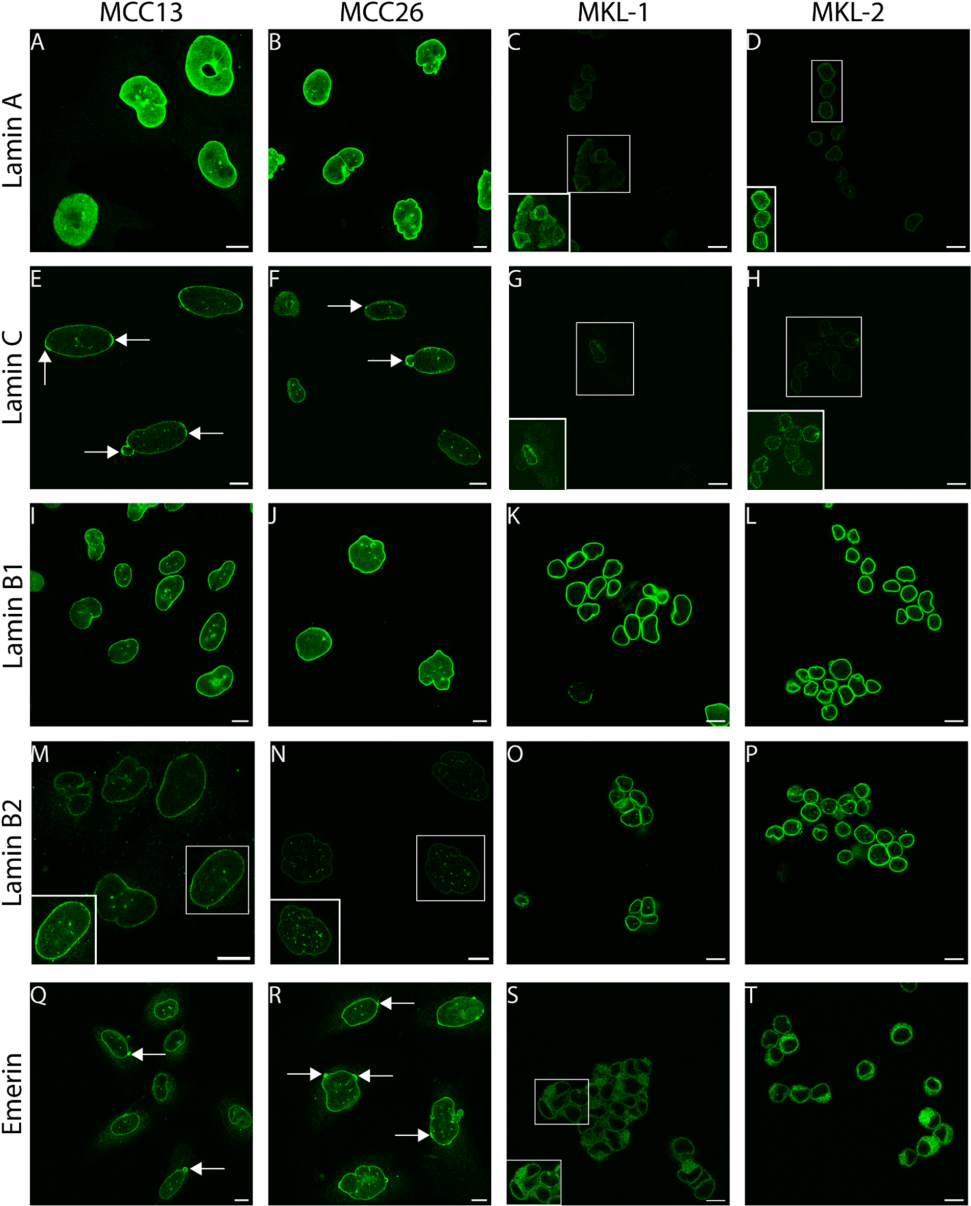
Differential lamin expression patterns in MCPyV-negative cell lines MCC13 and MCC26 as compared to MCPyV-positive cell lines MKL-1 and MKL-2. CLSM images of lamin A (**A**–**D**), lamin C (**E**–**H**), lamin B1 (**I**–**L**), lamin B2 (**M**–**P**), and emerin (**Q**–**T**) immunostaining in MCC13 (**A**,**E**,**I**,**M**,**Q**), MCC26 (**B**,**F**,**J**,**N**,**R**), MKL-1 (**C**,**G**,**K**,**O**,**S**), and MKL-2 (**D**,**H**,**L**,**P**,**T**). Arrows in (**E**,**F**,**Q**,**R**) indicate inhomogeneous distributions of lamins or emerin. Cells present in ROIs in **C**,**D**,**G**,**H**,**M**,**N** are displayed at enhanced brightness in the lower left corner of the same image. Scale bars: 10 μm.

Immunofluorescence stainings of both A- and B-type lamins were recorded with identical CLSM settings for each image to allow a (semi)quantitative comparison (Figs. 1 and Supplemental Figure S3). A-type lamin expression is significantly lower in MCPyV-positive cell lines as compared to MCPyV-negative cell lines (Figs. 1A-H, Supplemental Figures S3A, B). In addition, the immunoreactivity patterns for lamin C were found to be weaker than those for lamin A. Evaluation of the B-type lamins demonstrates significant differences in lamin B1 expression levels in MCPyV-negative and MCPyV-positive cell lines, with lower lamin B1 expression in MPCyV-negative cells (Figs. 1I-L and Supplemental Figures S3C), but the magnitude of this difference is less pronounced when compared to the differences seen for A-type lamins. For lamin B2, expression levels are also lower in the MCPyV-negative cell lines (Fig. 1M-P and Supplemental Figures S3D). Interestingly, the lamin stainings of the MCPyV-negative cells reveal abnormal nuclear morphologies (see for example Fig. 1A and J). Moreover, the lamin C staining displays an uneven distribution in the nuclear lamina, with higher fluorescence intensities particularly in nuclear herniations (arrows in Fig. 1E and F).

Immunofluorescence staining patterns of emerin reveal an inhomogeneous distribution in the NE of the MCPyV-negative cells, including nuclear herniations showing a more intense staining (arrows in Fig. 1Q, R), and a largely cytoplasmic (most likely endoplasmic reticulum) localization in the MCPyV-positive cells (Fig. 1S, T).

The intensity of the lamin immunostainings was quantified in both the nuclear lamina and the nucleoplasm (Supplemental Figure S3). Comparing the ratio (Supplemental Table S1) of these between the MCPyV-negative and -positive cells for A-type lamins demonstrates a low ratio in the lamin C staining of the MKL-1 cell line, which results from a very weak lamin signal in the nuclear rim (Fig. 1G and Supplemental Figure S3B). The ratio is higher for B-type lamin in the MCPyV-positive cells as compared to the MCPyV-negative cells, especially for lamin B1, which is due to an increased signal at the nuclear lamina and lower nucleoplasmic lamin B1 signal. Lamin B2 localization at the nuclear lamina is lower in the MCPyV-negative cells.

These data suggest differences in both A- and B-type lamin protein expression between MCPyV-negative and -positive cell lines, with lower expression of A-type lamins and higher expression of B-type lamins in the MCPyV-positive cell lines. Moreover, an aberrant nuclear morphology and inhomogeneous localization of lamins C and emerin can only be observed in the MCPyV-negative cells. In the MCPyV-positive cell lines, emerin is largely re-localized to the endoplasmic reticulum, due to the low levels of A-type lamins in these cells, as shown in previous studies^75,76^.

#### Western blotting

**Fig. 2:**
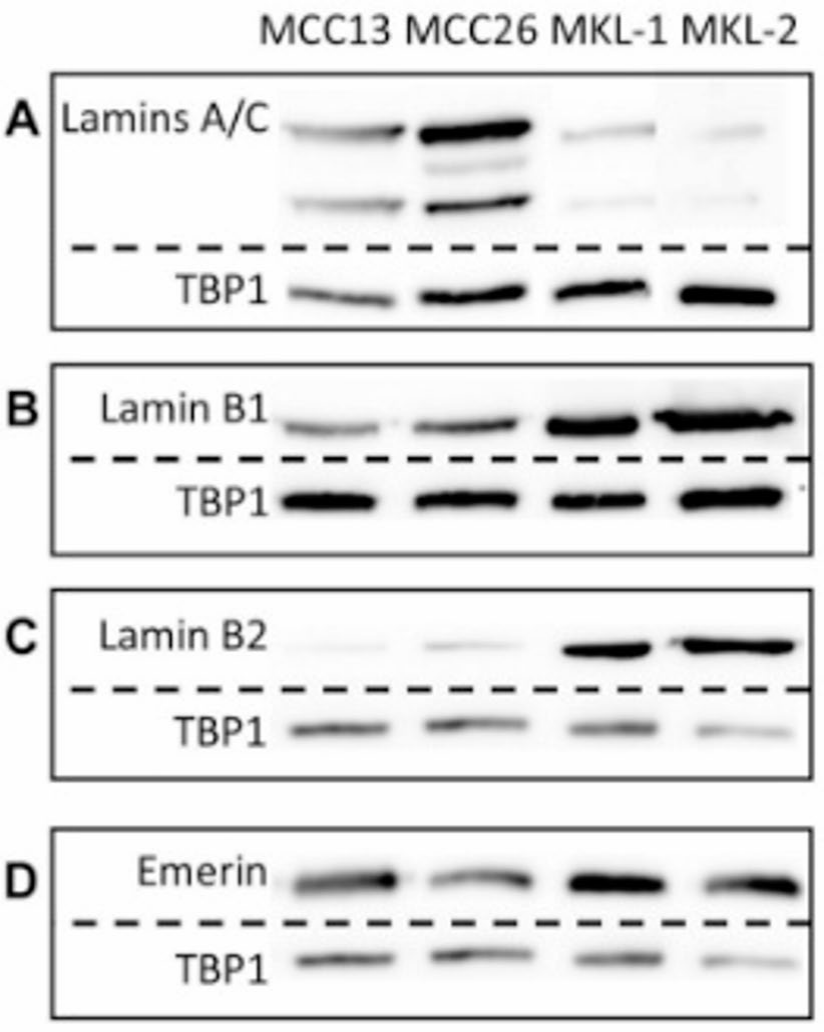
Western blotting showing differential A- and B-type lamin expression in MCPyV-negative cell lines MCC13 and MCC26, as compared to MCPyV-positive cell lines MKL-1 and MKL-2. (**A**–**D**) Western blotting result using antibodies against lamins A and C (**A**), lamin B1 (**B**), lamin B2 (**C**), and emerin (**D**). An antibody against TBP1 (molecular weight 38 kDa) was used as a loading control. Cropped blots are shown, original blots are presented in Supplemental Figure S4.

The differences in the immunofluorescence levels of the A-type lamins between the MCPyV-negative, adherently growing MCC13 and MCC26 cell lines, and the MCPyV-positive, non-adherent cell lines MKL-1 and MKL-2 were confirmed by the Western blotting data when using the Jol2 antibody that recognizes both lamins A and C (Fig. 2A). The results show that both A-type lamins are very weakly detected in the MKL-1 and MKL-2 immunoblots, while a positive reactivity for both A-type lamins is observed in MCC13 and MCC26. The opposite is true for the B-type lamins (Fig. 2B and C), showing a positive reactivity in the MKL-1 and MKL-2 immunoblots, while MCC13 and MCC26 show lower expression of the B-type lamins. Furthermore, emerin is expressed at similar levels in all four cell lines (Fig. 2D).

#### RT-qPCR

**Fig. 3:**
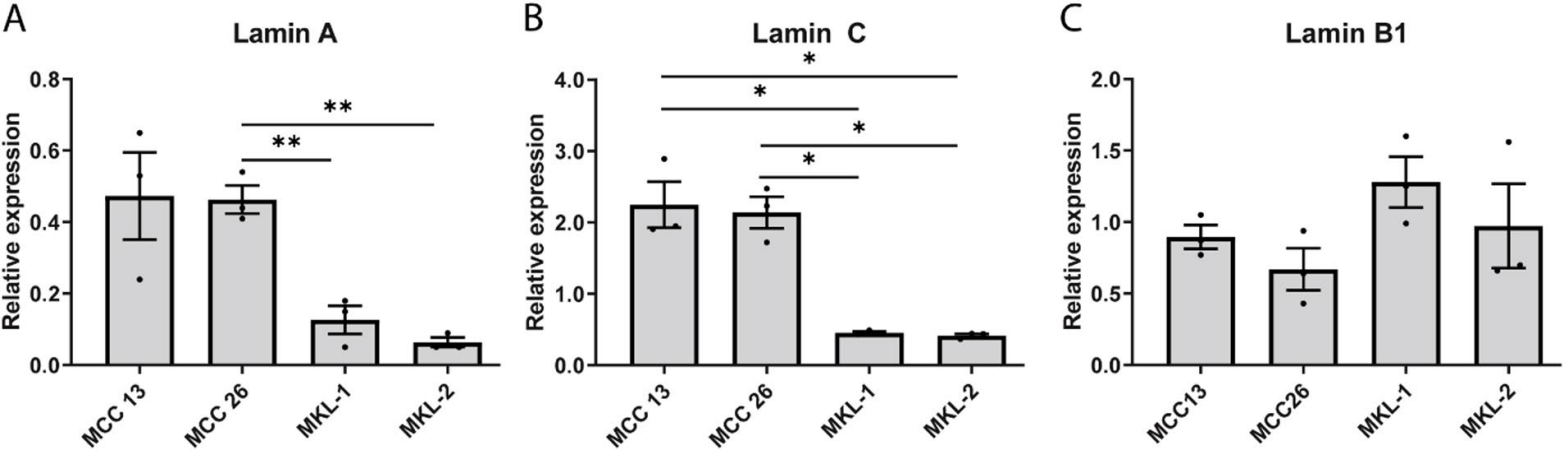
mRNA levels of A- and B-type lamins in the MCPyV-negative cell lines MCC13 and MCC26, compared to those of the MCPyV-positive cell lines MKL-1 and MKL-2, as determined by RT-qPCR. **A**) Lamin A; **B**) Lamin C; **C**) Lamin B1. The expression values were calculated relative to the housekeeping gene HPRT. Data is represented as mean ± SD and each value is plotted separately. *N* = 3, each value represents the average of triplicates. *p ≤ 0.05, **p ≤ 0.01. The significance levels between the different cell lines were as follows: Lamin A: MCC26 vs. MKL-1 *p* = 0.004, MCC26 vs. MKL-2 *p* = 0.005. Lamin C: MCC13 vs. MKL-1 *p* = 0.03, MCC13 vs. MKL-2 *p* = 0.03, MCC26 vs. MKL-1 *p* = 0.02, and MCC26 vs. MKL-2 *p* = 0.02.

To further substantiate the differential lamin protein expression in MCPyV-negative and -positive cells, the A- and B-type lamin mRNA levels were determined using RT-qPCR. These data reveal a significant reduction of both lamin A and lamin C mRNA levels in MCPyV-positive cells as compared to MCPyV-negative cells (Fig. 3A, B), which is in line with the findings of the protein expression studies. Lamin B1 mRNA levels are lower, although not significantly, in MCPyV-negative cell lines than in MCPyV-positive cell lines (Fig. 3C), as is also clearly seen at the protein level with Western blotting (Fig. 2B), but less apparent in the immunofluorescence study (Fig. 1I-L). For lamin B2, a very low mRNA level was found in all four cell lines, which was even undetectable for MKL-2, also at lower cDNA dilutions than used for lamins A, C, and B1.

Collectively, the immunofluorescence staining, Western blotting, and RT-qPCR findings, show lower A-type lamin mRNA transcription and protein expression in MCPyV-positive cells as compared to MCPyV-negative cells. In addition, a slightly higher lamin B1 expression level was found at both mRNA and protein level. For lamin B2, an enhanced level of protein expression is detected in the MCPyV-positive cells in immunofluorescence staining and Western blotting, but this could not be confirmed by qPCR due to very low mRNA detection levels in all four cell lines.

### Local accumulations of lamin C and emerin in the nuclear lamina of MCPyV-negative cell lines

**Fig. 4:**
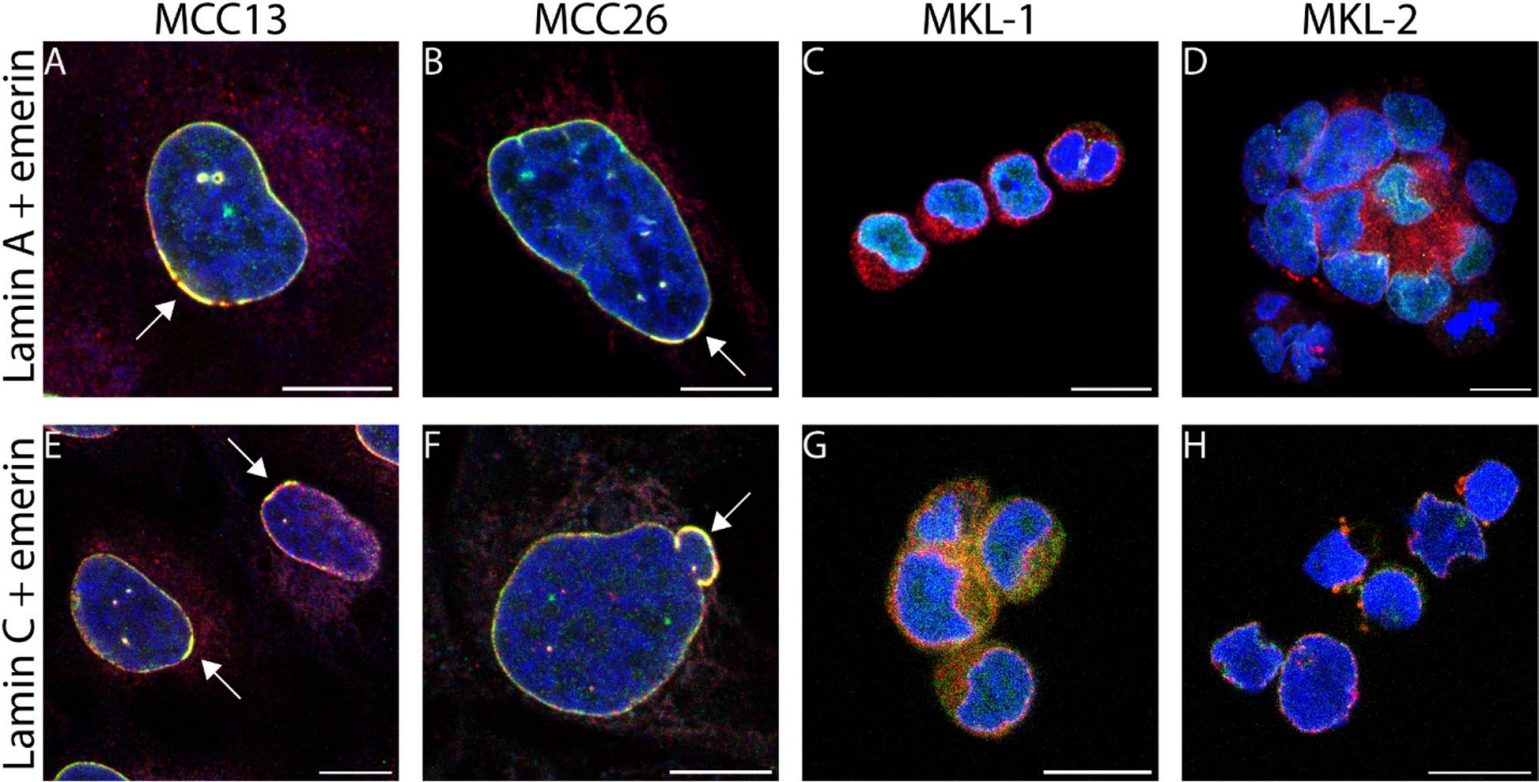
Local accumulation of lamin A and lamin C and re-localization of emerin in MCC cell lines. (**A**–**D**) Merged CLSM images of emerin (red), lamin A (green), and DAPI (blue) in MCC13 (**A**), MCC26 (**B**), MKL-1 (**C**), and MKL-2 (**D**). (**E**–**H**) Merged CLSM images of emerin (red), lamin C (green), and DAPI (blue) in MCC13 (**E**), MCC26 (**F**), MKL-1 (**G**), and MKL-2 (**H**). Emerin labelling demonstrates the main localization at the NE in MCPyV-negative cell lines, but with local accumulations (see arrows in **A**,**B**,**E**,**F**), while cytoplasmic localization, most likely representing emerin immunostaining in the endoplasmic reticulum, is seen in the MCPyV-positive cell lines (**C**,**D**,**G**,**H**). Both lamin A and lamin C show overlapping local accumulations with emerin. The brightness of the images was enhanced to illustrate the differences in lamin and emerin distribution. For non-merged images see Supplemental Figures S8 and S9. Scale bars: 10 μm.

Since the presence of A-type lamins in the nuclear lamina is critical for correct emerin localization at the nuclear lamina^75,76^, the correlation between irregularities in A-type lamin and emerin localization irregularities has been examined in more detail using double immunofluorescence labelling for emerin and either lamin A or lamin C (Fig. 4, Supplemental Figures S8 and S9). In MCPyV-negative cells overlapping local emerin and lamin A or C accumulations at the NE are visible in both normal appearing cells as well as in cells with nuclear abnormalities (Fig. 4A, B, E, F). In contrast, emerin localization in MCPyV-positive cells was merely cytoplasmic rather than nuclear, most likely representing relocalisation of emerin to the endoplasmic reticulum in the absence of A-type lamins, as described earlier (Figs. 4C, D, G, H). Indeed, as shown above, the MCPyV-positive cells demonstrated a low level of A-type lamins, without abnormal nuclear morphology and no obvious lamin accumulations.

### Morphological abnormalities in nuclei of MCPyV-negative cell lines

**Fig. 5:**
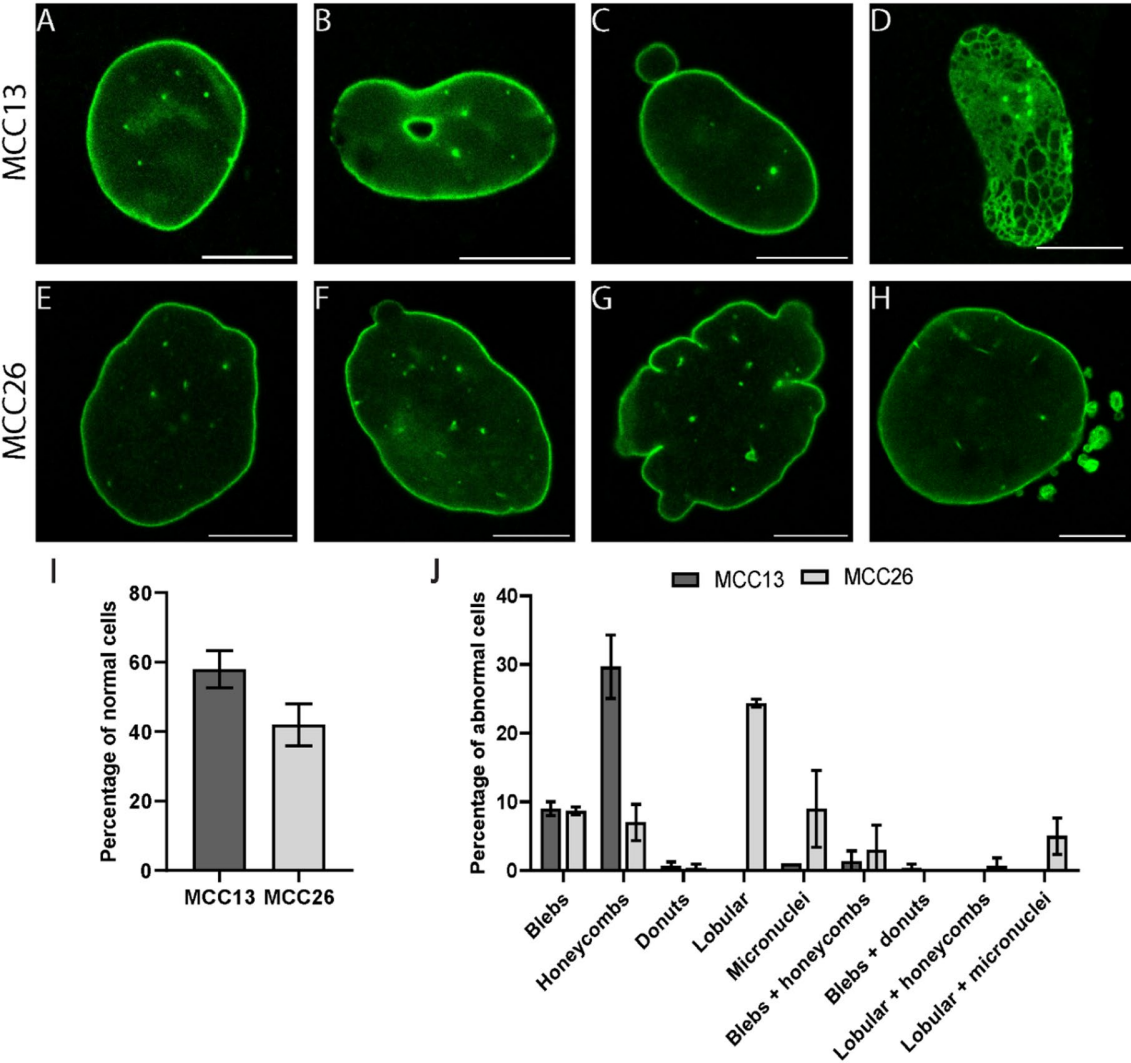
Nuclear aberrations in MCPyV-negative cell lines MCC13 (**A**–**D**) and MCC26 (**E**–**H**) visualized using anti-lamin B1 immunostaining (**A**–**C**,**E**–**G**) and anti-lamin A immunostaining (**D**,**H**). Images were acquired with CLSM and single z-slices are shown. Next to a normal nuclear morphology (**A**,**E**), different morphological abnormalities are seen, such as donut-shaped nuclei (**B**), micronuclei (**C**,** H**), honeycomb-like structures (**D**), blebs (herniations) (**F**), and lobular structures (**G**). The frequency of normal (**I**) and different abnormal (**J**) nuclear morphologies is displayed as mean percentages ± SD (average of *n* = 3 of 100 cells each, counting 300 cells in total). Scale bars: 10 μm.

The immunofluorescence studies revealed abnormal nuclear morphologies in MCPyV-negative cell lines, which could be detected after immunostaining for lamin B1 (Fig. 5A-C and E-G) and lamin A (Fig. 5D and H). These aberrations could not be observed in MCPyV-positive cell lines. Therefore, the frequency and type of nuclear abnormalities were assessed and quantified in lamin B1 immunolabelled MCC13 and MCC26 cells (Fig. 5I, J). These results indicate a high frequency of nuclear abnormalities, as only 58 ± 5% of MCC13 and 42 ± 6% of MCC26 cells have a normal nuclear morphology, as defined by nuclei with a regular round to oval shape and a homogenous lamina staining^36^ (Fig. 5A, E, I). In MCC13, the most frequently observed abnormalities were honeycomb-like lamin structures, whereas in MCC26, lobular-shaped nuclei were the most common (Fig. 5J). Strikingly, lobular nuclei were not detected in MCC13. Furthermore, both cell lines contained nuclei with blebs, donut-shaped nuclei, and micronuclei. Also, combinations of different morphological abnormalities were found. These results clearly demonstrate the presence of abnormalities in the nuclear morphology in MCPyV-negative cell lines, which resemble those seen in laminopathy cells. However, *LMNA* mutations or variants have not been detected in the MCC13 and MCC26 cell lines with Sanger sequencing.

### Occurrence of reversible NE ruptures in MCPyV-negative cell lines

**Fig. 6:**
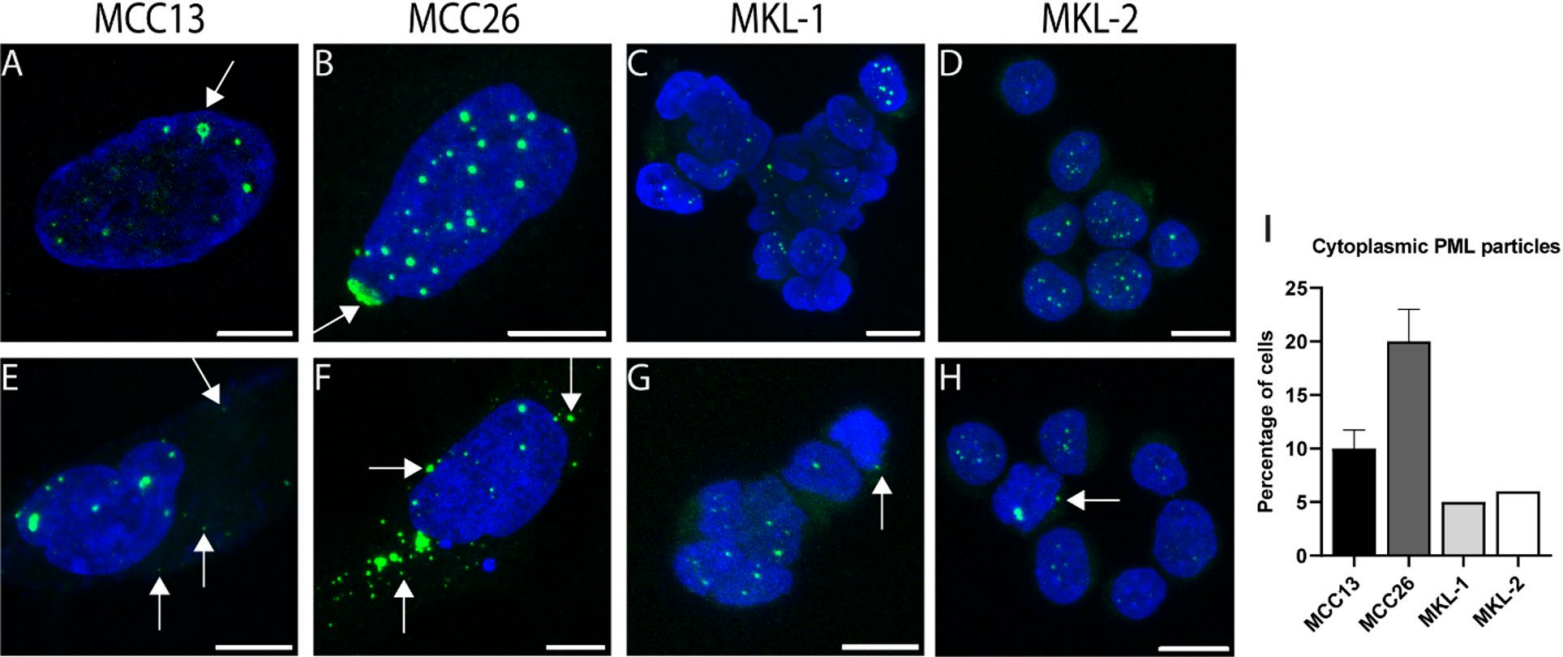
Localization of PML NBs. (**A**–**H**) Maximal z-projections of CLSM images of PML immunostaining (green) counterstained with DAPI (blue) in MCC13 (**A**,**E**), MCC26 (**B**,**F**), MKL-1 (**C**,**G**), and MKL-2 (**D**,**H**). Arrows indicate aspecific PML NB formations in **A**,**B** and cytoplasmic PML particles in (**E**–**H**). (**I**) The percentage of cells containing cytoplasmic localized PML NBs. Data is represented as mean ± SD. MCC13 and MCC26: average of *n* = 3 with 100 cells each, counting 300 cells in total. MKL-1 and MKL-2: *n* = 1 measurement of counting 100 cells. Scale bars: 10 μm.

Since an abnormal lamin distribution and aberrant nuclear morphology can have consequences for nuclear integrity and can, for instance, lead to NE ruptures, the occurrence of nuclear ruptures was investigated in both the MCPyV-negative and MCPyV-positive cell lines. First, the dislocation of Promyelocytic Leukemia Nuclear Bodies (PML NBs) into the cytoplasm was assessed, since this can be used as a surrogate marker in fixed cells for NE ruptures^77^. The CLSM images revealed a higher frequency of cytoplasmic PML particles (PML CPs) in MCPyV-negative cells as compared to MCPyV-positive cells, with the highest frequency in MCC26 (Fig. 6). Strikingly, the nuclear localized PMLs in the MCPyV-negative cells occasionally demonstrated aberrant PML NB formations, such as ring- and fusion-like structures (arrows in Fig. 6A, B).

**Fig. 7:**
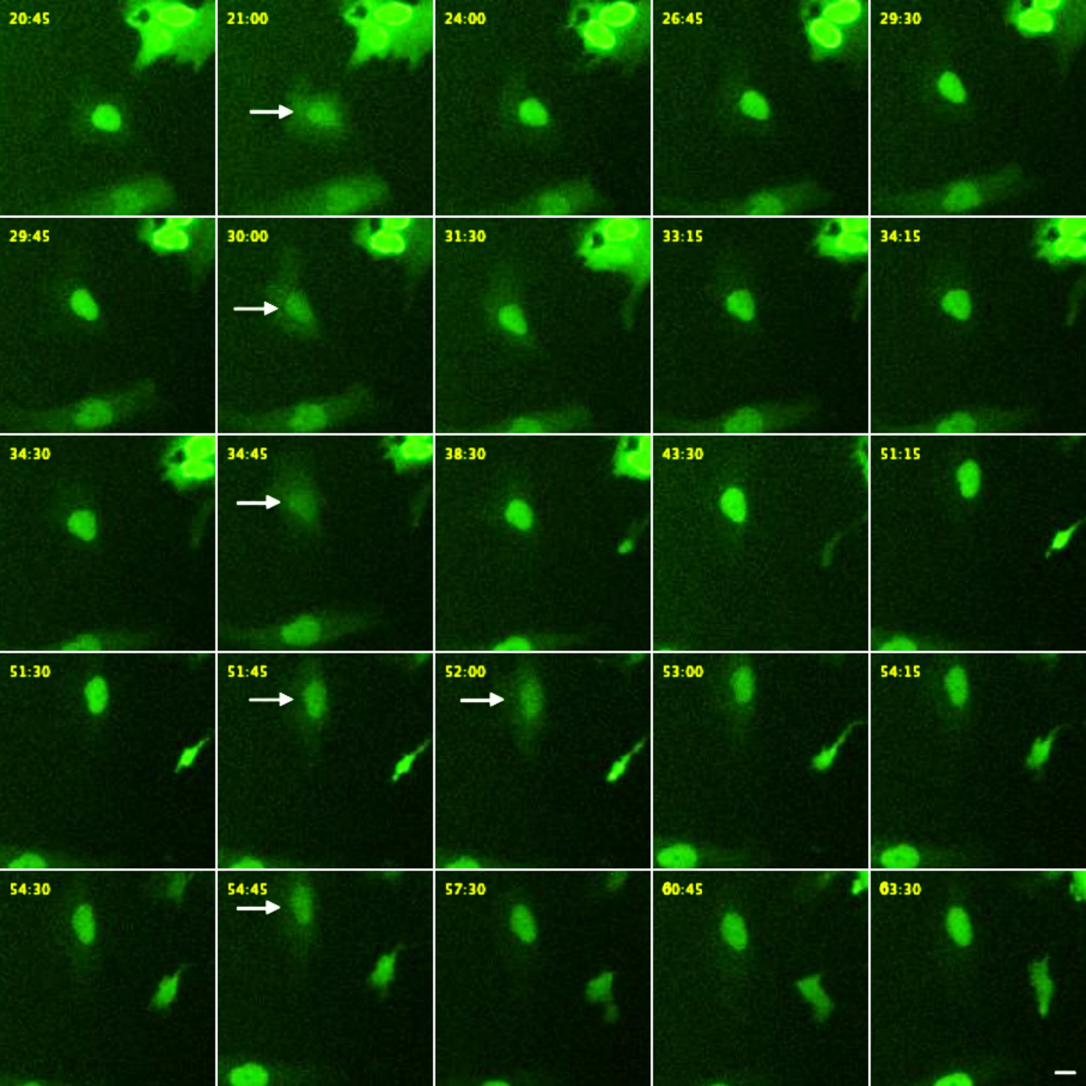
Repetitive nuclear envelope (NE) ruptures in MCC13 cells. Selected images from a recording using the IncuCyte Zoom imaging system (Supplemental Video S1 and S2) of MCC13 cells transfected with the construct encoding NLS-EYFP. Note 5 consecutive ruptures within one hour in the same cell. Images at the left show the cell just before rupture. Arrows in the second row from left point to the NE rupture event, while (partial) restoration of the nuclear signal is visible in the following columns to the right, indicating closure of the NE and survival of the cell. Time passaged in minutes since initiation of recording is indicated at the top left corner. Scale bar represents 10 μm.

**Fig. 8:**
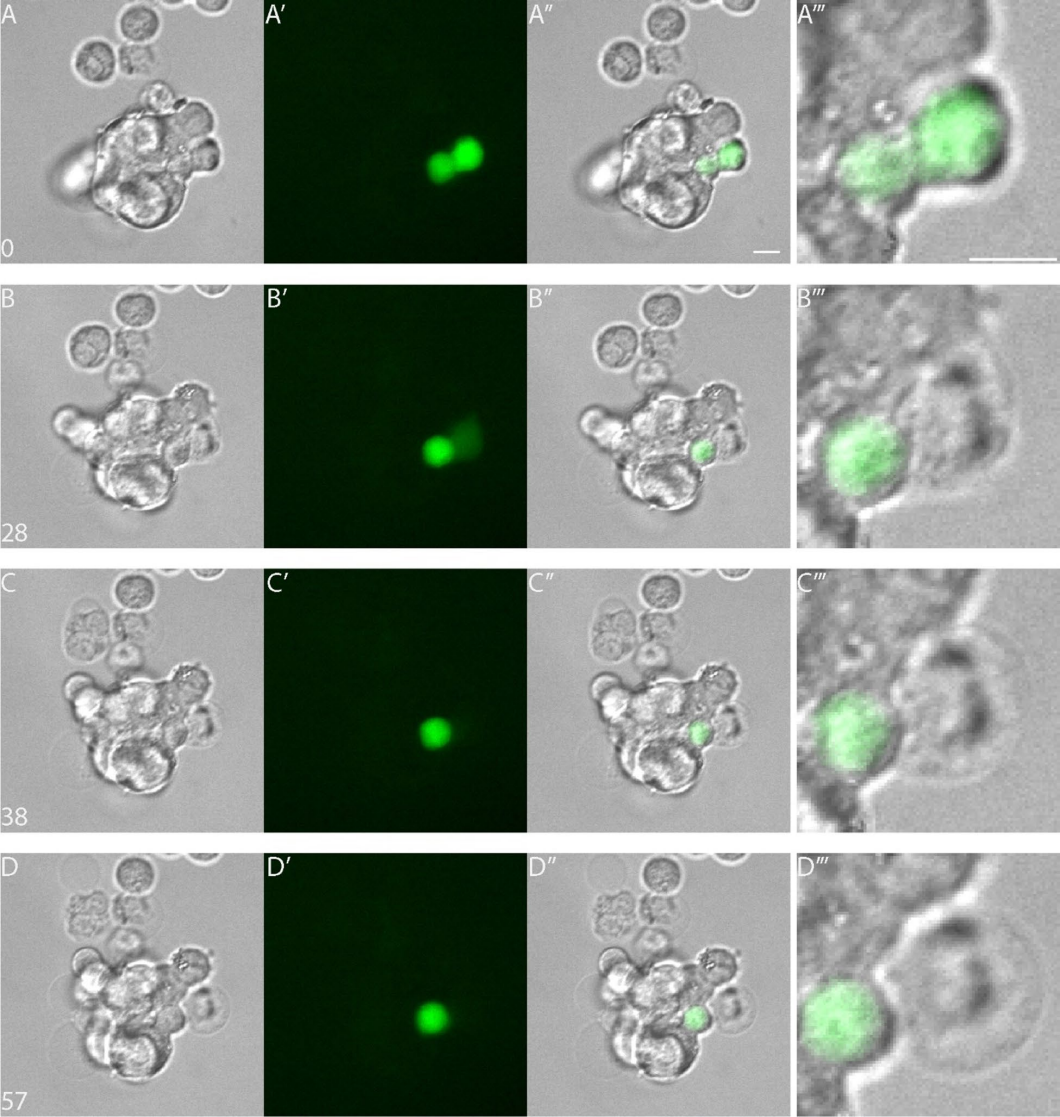
Nuclear envelope (NE) rupture and cell death in MKL-2. Selected images from a recording using the vital imaging system (Supplemental Video S3) of MKL-2 cells transfected with the construct encoding NLS-EYFP. Differential Interference Contrast (DIC) images (**A**–**D**), fluorescence images (**A’**–**D’**), and merged images (**A’’**–**D’’**) with magnification of selected region (**A’’’**–**D’’’**) demonstrate that NE rupture occurs at 28 min after initiation of recording, resulting in induction of cell death as concluded from the total disappearance of the EYFP signal from the cell and its translucent appearance and swollen cellular membrane at t = 57 min. Scale bars represent 10 μm.

Because the presence of PML CPs suggests the occurrence of NE ruptures, cells were transfected with an expression vector encoding a nuclear localization signal tagged with EYPF (NLS-EYFP) and subsequently subjected to live cell imaging. Time-lapse recordings of MCPyV-negative cells demonstrated the occurrence of numerous repetitive NE ruptures, as seen by the abrupt (partial) loss of the nuclear EYFP signal, which gradually re-appeared within the following minutes to hours (Fig. 7, Supplemental Figure S10, and Supplemental Videos S1 and S2; for similar results see^26^, suggesting repair of the NE. MCPyV-positive cells also exhibited NE ruptures (Fig. 8, Supplemental Figure S11, and Supplemental Videos S3 and S4), but these seem not to be repaired, since no reappearance of a nuclear EYFP signal can be detected in these cells. Rather, these MCPyV-positive cells seem to undergo cell death, based on the disappearance of the EYFP signal from the cell and its translucent appearance and swollen cellular membrane, as visible in the higher magnification in Fig. 8D’’’.

These results indicate the occurrence of NE ruptures in both the MCPyV-negative and -positive cell lines, but while these seem to be irreversible and lethal in MCPyV-positive cells, they are reversible in MCPyV-negative cells.

A comparison between properties of MCPyV-negative and MCPyV-postive cells is summarized in Table 1.

**Table 1:** Overview of observed differences between MCPyV-negative (MCC13, MCC26) and MCPyV-positive (MKL-1, MKL-2) cell lines in lamin expression levels, Emerin localization, nuclear abnormalities and nuclear envelope (NE) ruptures. The lamin expression levels are compared between the MCPyV-negative and MCPyV-positive cell lines and based on this difference designated as low, high, or moderate. Protein expression levels are based on Immunofluorescence staining intensities and Western blotting. mRNA expression levels are based on results of RT-qPCR.

Cell line	MCPyV-negative			MCPyV-positive		
	MCC13	MCC26		MKL-1	MKL-2	
Lamin A expression						
*Protein level*	High Local accumulations	High Local accumulations		Low	Low	
*mRNA level*	High	High		Low	Low	
Lamin C expression						
*Protein level*	High Local accumulations	High Local accumulations		Low	Low	
*mRNA level*	High	High		Low	Low	
Lamin B1 expression						
*Protein level*	Moderate	Moderate		High	High	
*mRNA level*	Moderate	Moderate		Moderate/high	Moderate/high	
Lamin B2 expression						
*Protein level*	Low	Low		High	High	
Emerin localization	NE with local accumulations	NE with local accumulations		Largely cytoplasmic (ER)	Largely cytoplasmic (ER)	
Nuclear abnormalities	Yes	Yes		No	No	
NE ruptures	Yes, NE repair observed	Yes, NE repair observed		Yes, NE repair not observed	Yes, NE repair not observed	

### Effects of MCPyV-sT- and LT antigen on lamin expression in MCPyV-negative cell line MCC13

In order to investigate whether the differential A-and B-type lamin expression between MCPyV-negative and -positive cells are caused by the viral integration of MCPyV, the MCPyV-negative cell line MCC13 was transfected with constructs encoding the viral antigens MCPyV-sT (MCC13sT) and MCPyV-LT (MCC13LT). The constructs also encode GFP as a positive transfection control. Transfection with an empty vector (MCC13EV), a construct which does not code for MCPyV T-antigens but only for GFP, was performed to evaluate the potential effect of the transfection process. First, the presence of MCPyV-sT and MCPyV-LT was confirmed by RT-qPCR^78^ and by immunofluorescence staining for MCPyV-LT (Supplemental Figure S12). Additionally, CK20 expression was not altered upon transfection, as demonstrated by negative immunofluorescence staining (Supplemental Figure S13) and transcript quantification (Supplemental Figure S14).

**Fig. 9:**
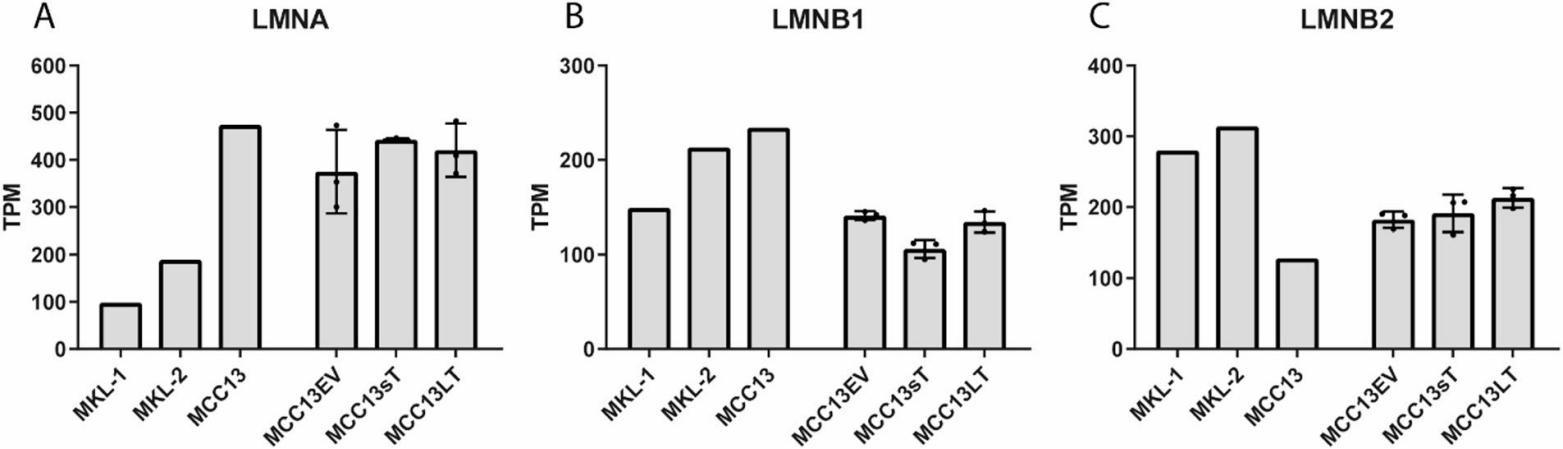
Expression levels (transcripts per million; TPM) of lamins A/C (LMNA), lamin B1 (LMNB1), and lamin B2 (LMNB2) based on RNA sequencing of MCPyV-positive cell lines MKL-1 and MKL-2 and in MCPyV-negative cell line MCC13, either non-transfected or transfected with the construct encoding MCPyV-LT (MCC13LT), MCPyV-sT (MCC13sT), or an empty vector (MCC13EV). MCC13LT, MCC13sT, and MCC13EV were analyzed in triplicate, data represent mean ± SD and each value is plotted separately.

RNA sequencing analysis of MCC13sT and MCC13LT did not demonstrate substantial differences in *LMNA*, *LMNB1*, and *LMNB2* expression levels as compared to MCC13EV or non-transfected MCC13 (Fig. 9). Immunofluorescence stainings of non-transfected MCC13 and transfected MCC13 also did not reveal similar changes in lamin expression levels as seen when comparing MCPyV-positive and -negative cells (Supplemental Figure S15). The abnormal nuclear morphologies and lamin scars seen in MCC13 were still visible after transfection, indicating that the nuclear lamina structure was unaltered in transfected cells. Taken together, these data demonstrate that the expression of MCPyV-sT or MCPyV-LT alone is not sufficient to downregulate the A-type or upregulate the B-type lamin expression levels in MCC cells.

## Discussion

MCC has a high propensity for metastasis; at the time of diagnosis, about 34% of the patients have regional lymph node or distant metastases^79^. Differential lamin expression is reported in a growing number of cancers and levels of lamin expression are suggested to be indicative of the clinical behavior of tumors and their metastatic potential^43,50,54,55,80–82^. The underlying study therefore examined A- and B-type lamin expression in MCPyV-positive and -negative MCC cell lines and its effect on cell behavior.

For this study, MKL-1 and MKL-2 were used as MCPyV-positive cell lines and were found to be positive for the MCC marker CK20, while the MCPyV-negative cell lines MCC13 and MCC26 were negative, which confirms previous findings^5^. MCC13 and MCC26 have been described as variant MCC lines and may not be fully representative of MCC tumors^83,84^. MCPyV-positive cell lines demonstrated lower A-type lamin expression level as compared to MCPyV-negative cell lines, as demonstrated by using several molecular methods. To further investigate the potential effect of MCPyV on differential lamin expression, the MCPyV-negative cell line MCC13 was transfected with constructs encoding MCPyV-sT or MCPyV-LT. The expression of one of these viral proteins did not induce a similar reduction of A-type lamin expression. Yet, this does not exclude the possibility that MCPyV integration can affect lamin expression, since in our transfection studies, the viral genome is not integrated into the host DNA. This integration may trigger signaling pathways affecting lamin expression, possibly resulting in a downregulation of A-type lamins as seen in MKL-1 and MKL-2. Alternatively, multiple factors may be required, such as the simultaneous expression of MCPyV-sT and MCPyV-LT, similar to the need for both viral proteins pUL34 and pUL31 for nuclear lamina disruption in herpes simplex 1 (HSV-1) infection^85^. It should also be noted that MCPyV-mediated carcinogenesis requires a truncated form of LT^14^.

The difference in A-type lamin expression between MCPyV-positive and MCPyV-negative cells is more likely to be due to the difference in their adhesion capacity. Cells that grow in suspension cultures have a low cell tension^86^, whereas a stiff growth environment (such as a glass slide or plastic bottom of a culture dish) is correlated with higher lamin A levels, as also found in a study into lamin A expression levels of cells grown on soft and stiff matrices^87^. A study using breast cancer cells demonstrated increased lamin A/C expression levels upon reattachment of cells to the culture dish after suspension culture^86^. Low levels of A-type lamins were previously found in SCLC^51^, which is also a neuro-endocrine tumor type and has morphological similarities to MCC^88^. Most SCLC cell lines also grow in suspension, similar to MCPyV-positive MCC cells, and the low A-type lamin expression patterns in these SCLC cell lines are similar to the low levels in SCLC patient tissue samples^51^. When introducing the v-rasH oncogene into the non-adherent NCI-H249 SCLC cell line, these cells become surface adherent^89^ and as a result a greater than 10-fold increase in the amounts of lamins A and C could be observed^90^.

Collectively, these studies demonstrate that the growth behavior of cells and A-type lamin expression levels are correlated. The distinct growth behavior of MCPyV-positive and -negative MCC cell lines might be related to their MCPyV status. In a small study of MCC tissues, MCPyV-positivity was found to be inversely correlated with the expression of β5-integrin, an adhesion molecule that mediates the interaction with the extracellular matrix^91^. Also, in MCC cell lines, the β5-integrin expression was found to be high in adhering MCC13 and MCC26, while this was absent in non-adhering MKL-1 and MKL-2^91^. Studies using MCPyV-sT transfection in HEK cells revealed that the sT antigen seems to be responsible for increased cell motility, which was triggered by reduced integrin β1 phosphorylation^92^. Lamins are linked to integrins via the LINC complex and tension from integrins was found to be communicated through this complex to the nuclear lamina^93^. This leads to the hypothesis that the presence of integrated MCPyV, and most likely the expression of MCPyV-sT, lowers integrin levels or their potential to interact with the extracellular matrix, which releases tension on the nucleus and lowers A-type lamin expression levels. However, the expression of MCPyV-sT alone in MCC13 did not result in lower A-type lamin expression in MCC13sT. Further research into the underlying mechanism of low A-type lamin expression of MCPyV-negative cells is needed.

Next to A-type lamins, also B-type lamin expression was examined, showing that lamin B1 expression was higher in MCPyV-positive cells at both the mRNA and protein level. Lamin B2 expression also revealed higher expression at the protein levels in MCPyV-positive cells as compared to MCPyV-negative cells.

In addition to the differences in the lamin expression levels between MCPyV-positive and -negative cells, local accumulations of A-type lamins and emerin in the NE, as well as abnormal nuclear morphologies, were observed in MCPyV-negative cells. These local accumulations strongly resemble the sites of NE repair (also indicated as “lamin scars”), which can last for several hours after NE rupture^94^. Indeed, MCPyV-negative cells can exhibit numerous reversible NE ruptures within a few hours, indicating an efficient repair of the nuclear membrane during interphase. Previous research revealed that lamin C accumulates rapidly at the NE rupture site for repair^95^, while another study demonstrated the accumulation of emerin at NE rupture sites, even in the absence of lamins A/C^96^, . However, knockdown of lamins A and C slows down barrier-to-autointegration factor (BAF) and cytoplasmic cyclic GMP-AMP synthase (cGAS) accumulation at the NE rupture site, other important components for NE repair^95^. Similarly, silencing of emerin mRNA also increases the time needed for NE rupture repair^96^. The accumulation of A-type lamins and emerin found in the MCPyV-negative cells therefore suggests the recent occurrence of a NE rupture and ongoing local repair of the NE. In MCPyV-positive cells NE ruptures do not seem to be restored, resulting in cell death. This can be explained by the fact that a general low level of A-type lamin expression is found in MCPyV-positive cells, resulting in a re-localization of emerin to the endoplasmic reticulum. The latter can be expected since lamin A is crucial to localize emerin correctly into the NE^75,76^. These findings strengthen the hypothesis that local accumulation of A-type lamins and emerin are a prerequisite for the repair of NE ruptures.

Striking similarities are found between MCPyV-negative cells and laminopathy cells. The higher number of PML particles in the cytoplasm of MCPyV-negative cells as compared to MCPyV-positive cells suggests a higher frequency of NE ruptures in MCPyV-negative cells. A high frequency of both PML CPs and NE ruptures are also reported in laminopathy dermal fibroblasts^77^. Also, the abnormalities in nuclear morphology seen in the MCPyV-negative cell lines resemble those seen in laminopathy cells. These abnormalities could not be observed in MCPyV-positive cell lines. In this respect it is of interest that Tamiello et al.^97^ have shown that laminopathy cells, demonstrating nuclear abnormalities when grown on a stiff substrate, no longer exhibit such abnormalities when grown on a soft substrate. Likewise, the low tension on the nucleus of MKL-1 and MKL-2 growing as non-adherent aggregates may be the reason for the absence of nuclear abnormalities in these cells.

We have not obtained any indications that *LMNA* mutations, as seen in laminopathy cells, occur in the MCC cell lines. It has been described that in general, MCPyV-negative MCC tumors and cell lines derived therefrom have a higher mutational burden and higher frequency of chromosomal copy number variations (CNVs) as compared to the MCPyV-positive MCC tumors and cell lines^98^. CNVs in MKL-1 have been investigated, but these have not been found for chromosomes that contain the lamin gens (i.e. chromosome 1 for *LMNA*, chromosome 5 for *LMNB1*, and chromosome 19 for *LMNB2*). Furthermore, other genome sequencing studies did not reveal any lamin mutations (including *LMNA*,* LMNB1*, and *LMNB2*) in MCC13, MCC26, MKL-1, and MKL-2^99,100^. In addition, in a cohort of 464 cases of MCC solid tumors (primary and metastases) no *LMNA*,* LMNB1*, or *LMNB2* mutations were detected^101^. It should be kept in mind, however, that the NE ruptures frequently seen in the MCC cell lines results in genomic instability through the loss of chromatin from the nucleus^94^.

Possibly the abnormal nuclear morphology in the MCPyV-negative cell lines is due to one or more mutations in other NE-associated proteins, such as nesprin-1, which is mutated in both MCC13 and MCC26^99,100^.

In laminopathy cells expressing mutated A-type lamins, NE ruptures were also found to correlate with nuclear aberrations^102^, while no direct correlation exists between the occurrence of PML CPs and nuclear aberrations^77^. Furthermore, selective knockdown, as well as knockout of *Lmna* in mouse fibroblasts has been demonstrated to increase the number of NE ruptures^102^. The lack of A-type lamins is therefore most probably the leading cause for NE rupture in MCPyV-positive cells. Additionally, the loss of tumor suppressors Rb and/or p53 can induce NE rupture^103^, which can occur in both MCPyV-positive cells and MCPyV-negative cells, although by different mechanisms^13^. The lack of NE rupture repair in MCPyV-positive cells and resulting cell death may possibly explain the less aggressive behavior of these cases as compared to the MCPyV-negative tumors^9,10^. Indeed, in breast cancer, an invasive phenotype was correlated with the occurrence of NE ruptures^104^.

Taken together, the A-type lamin expression in MCPyV-positive MCC cells is lower as compared to MCPyV-negative cells, while B-type lamin expression is (slightly) higher. The difference found in A-type lamin expression in the cell lines in this study is likely caused by their different growth patterns, while the mechanism that causes the differential growth behavior remains to be elucidated. However, regardless of the cause for the (non-)adherent growth in cell culture, differential A-type lamin expression, the frequency of NE ruptures, and the (lack of) repair thereof are relevant in the context of tumor growth and metastasis. Previous research has related low A-type lamin expression to high Paclitaxel sensitivity^105^. Paclitaxel is currently not used for treatment of MCC, as its clinical trial was not executed due to a lack of participating patients^106^, but these findings suggest that low A-type lamin expression is a therapeutic advantage.

Further research on these cell lines should focus on unravelling the impact of NE rupture and NE repair in cancer development. This can be done with migration assays to assess the potential impact of the NE ruptures on nuclear integrity as well as genome content of individual tumor cells, and their impact on tissue invasion and metastasis. If NE rupture and repair is an important contributor to the aggressive behavior of MCC, it is of interest to modify NE rupture repair as a therapeutic target. As NE ruptures are common in malignancies, elucidating the NE rupture repair machinery in more detail is of high importance to the field of cancer biology in general.

## Materials and methods

### Cell culture

#### MCPyV-positive and -negative cell lines

Two MCPyV-positive MCC cell lines MKL-1 (ECACC 09111801) and MKL-2 (https://www.cellosaurus.org/CVCL_D027) and two MCPyV-negative MCC cell lines MCC13 (ECACC 10092302) and MCC26 (ECACC 10092304) were used in these studies^107^. MKL-1 and MKL-2 cell lines grow non-adherent, whereas MCC13 and MCC26 are adherent cell lines. The cells were cultured in RPMI 1640 medium (Gibco, Waltham, USA) with 10% fetal calf serum (FCS; Gibco), 1% L-glutamine (MP Biomedicals, Illkirch, France), and 50 µg/mL Gentamycin (Dechra, Northwich, UK) at 37 °C and 5% CO_2_ in a humidified incubator. The adhering cell lines MCC13 and MCC26 were passaged at 1:3 to 1:4 ratios at confluent cell growth, using trypsin (0.05% trypsin, 0.02% EDTA, and 0.02% glucose in Phosphate Buffered Saline (PBS)). The non-adhering cell lines MKL-1 and MKL-2 were passaged by splitting at 1:2 to 1:3 ratios. If the cellular density was too low for splitting, the RPMI medium was refreshed after 2–3 days without splitting.

#### Cell transfections with NLS-EYFP

To examine NE ruptures in both MCPyV-positive and MCPyV-negative cell lines, the cells were transfected with a construct encoding an NLS-EYFP (gift from dr. J. Goedhart, University of Amsterdam). The non-adherent cells were seeded on the day of transfection at a density of 1 × 10^5^ cells/mL per well in a 12-wells plate, whereas adherent cells were seeded on the day before transfection at a density of 5 × 10^4^ cells/mL in a 6-wells plate. A transfection complex was formed by adding 1 µg DNA to 50 µL serum-free DMEM in a polystyrene tube, followed by adding 2 µL FuGENE HD Transfection Reagent (Promega, Madison, USA) directly into the medium. The tube was briefly vortexed and incubated for 15 min at room temperature (RT). The transfection complex was added dropwise to the medium and the well plates were swirled to ensure its homogeneous distribution over the entire surface. After 4 hours (h) incubation (37 °C and 5% CO_2_ in a humidified incubator) with the transfection reagent, the culture medium was refreshed. The cells were cultured at 37 °C and 5% CO_2_ in a humidified incubator until imaging at 24 to 48 h after transfection.

#### Cell transfections with constructs encoding MCPyV-sT- and -LT antigens

Using a doxycycline inducible gene expression system, constructs encoding either sT or LT of MCPyV, in addition to GFP, were transfected into the MCPyV-negative cell line MCC13, with an empty vector (EV), only containing GFP, serving as a control. The transfection procedure is performed as described by Macamo et al.^78^ and all procedures were carried out in triplicate. Based on GFP expression levels, single cells were sorted using a BD/FACS Canto II flow cytometer (BD Biosciences, Franklin Lakes, USA). The transfection efficiency for sT and LT was assessed by RT-qPCR^78^, as well as immunofluorescence for LT. After selection, the transfected MCC13 cells were cultured as described above, but in the presence of doxycycline (0.5 µg/mL culture medium; Sigma-Aldrich, St. Louis, USA).

### Immunocytochemistry

Adherent cells were seeded onto 18 mm round glass coverslips in a 12-wells plate. After approximately 48 h, the cells were fixed with methanol (-20 °C, 10 min) or with 4% formaldehyde (RT, 15 min). For adherence of the non-adherent cell lines, 18 mm glass coverslips were coated with 0.01% poly-L-lysine for 15 min and air dried for 15–30 min. Next, approximately 1 × 10^6^ cells/mL were allowed to adhere onto the coated cover slips for 30 min and fixation was performed as described above. The cells were stored at 4 °C in PBS containing 0.01% sodium azide.

Prior to immunofluorescence (IF) staining, formaldehyde fixed cells were permeabilized in Triton X-100 (0.1% in PBS) for 15 min at RT, followed by washing with PBS (3 × 3 min). After permeabilization, primary antibodies directed against lamin A, lamin C, lamins A/C, lamin B1, lamin B2, emerin, PML NBs, and MCPyV-LT antigen were diluted in blocking buffer (PBS with 3% bovine serum albumin (BSA); for detailed information about the antibodies see Supplemental Table S2), applied onto the coverslips, and incubated for 1 h at RT. For the anti-CK20 staining, cells were incubated overnight at 4 °C with the antibody diluted in PBS with 3% BSA and 0.1% Triton-X-100 (Supplemental Table S2). The CK20 antibody reveals a minor dot-like non-specific staining in a control cell line (normal human dermal fibroblasts), which is not present when omitting the secondary antibody. After primary antibody incubation and washing with PBS (3 × 3 min), secondary antibodies as specified in Supplemental Table S3 were diluted in blocking buffer, applied onto the coverslips, and incubated for 1 h at RT. A final washing step with PBS (3 × 3 min) was performed before the coverslips were mounted on a microscopy glass slide with Tris-Glycerol DABCO mounting medium (90% glycerol, 20 mM Tris-HCl pH 8.0, 2% 1,4-di-azo-bicyclo-2(2,2,2)-octane; Merck, Darmstadt, Germany) containing 2% diamidino-2-phenylindole (DAPI; Sigma-Aldrich), and sealed with nail polish. The slides were stored at 4 °C until imaging.

### Fixed cell imaging and analysis

CLSM imaging of immunostained cells described in Sect. 2.1, and 2.3–2.5 was performed with a Leica SPE confocal with LAS AF software (Leica, Wetzlar, Germany) using an ACS APO 63x/1.30 oil lens, format of 1024 × 1024, and speed of 400 Hz.

For the lamin immunostainings of MKL-1, MKL-2, MCC13, and MCC26 (Sect. 2.1, 2.3 and 2.4) the FITC conjugates were excited at 488 nm, while its emission was detected at 500–600 nm. DAPI was excited at 405 nm and its emission detected at 418–536 nm. The images were recorded as one focal plane and subsequently 20 cells per cell line were analyzed using the Fiji open-source platform for biological image analysis^108^. The fluorescence intensity of the lamina and the nucleoplasm was separately quantified using the Measure tool and the ratio between these signals was determined. For statistical analyses of the lamina intensities, two-tailed Student’s t-tests assuming unequal variances were performed. *P*-values ≤ 0.05 were considered statistically significant. Exact *p*-values are indicated in the text and indicated in the figures as follows: * for *p* ≤ 0.05, ** for *p* ≤ 0.01, *** for *p* ≤ 0.001, and **** for *p* ≤ 0.0001.

For lamin staining in MCC13 transfected with MCPyV T antigens (Sect. 2.5) a secondary antibody conjugated to Alexa Fluor 568 was used, which was excited at 532 nm and emission was detected at 575–655 nm. The GFP signal was excited at 488 nm and emission was detected at 498–558 nm, DAPI at 405 nm and 430–500 nm, respectively. To determine the fluorescence intensity in Fiji, the outline of a nucleus was selected with the wand tracing tool after using the threshold method Mean^109^. Next, the Measure tool was used to determine the mean fluorescence signal intensity in this region of interest (ROI).

Nucleoplasmic or cytoplasmic localization of PML NBs was assessed by analyzing CLSM z-stacks (step size of 0.5 μm).

CLSM imaging of the double-labeled cells as described in Sect. 2.2 was performed with a Leica TSC SP8 STED microscope with LAS X software (Leica) using an HC PL APO CS2 100x/1.40 oil lens, format of 1024 × 1024, and speed of 400 Hz. The secondary antibody goat anti-mouse IgG1-Texas Red used for detection of emerin was excited at 595 nm and emission was detected at 600–660 nm. The secondary antibody for detection of lamin A (goat anti-mouse IgG3-FITC) or lamin C (swine anti-rabbit Ig-FITC) was excited at 488 nm and detected at 500–560 nm. For DAPI these settings were 405 nm and 410–500 nm, respectively. Controls did not show cross-reactivity of the goat anti-mouse IgG1 with the lamin A IgG3 primary antibody, nor for the goat anti-mouse IgG3 with the emerin IgG1. Additionally, no bleed-through was seen between the FITC and Texas Red channels.

Nuclear abnormalities of MCC13 and MCC26 were scored by counting three times 100 cells immunostained for lamin B1 using a fluorescent microscope (Leica DMRBE) equipped with a PL APO 63x/1.32–0.6 oil objective.

### Live cell imaging and analysis

Cells expressing NLS-EYFP were recorded within 24–48 h after transfection using a vital imaging system consisting of an inverted fluorescence microscope (Leica DM IRBE; Leica) equipped with a Hamamatsu digital camera (CA4742-95; Hamamatsu Photonics, Shizuoka, Japan), a polychrome II polychromator (TILL Photonics, Gräfelfing, Germany), and a PL FLUOTAR 20x/0.50 lens containing an objective heater. The software program Openlab (version 2.1.1, 1999; Improvision, Coventry, UK) was used to record Differential Interference Contrast (DIC) and fluorescence images (476 nm excitation, 510–560 nm emission filter).

Cells were recorded during 2 h with an interval of 60 s.

Alternatively, live cell imaging of NLS-EYFP expressing MCC cells, grown in 6-wells plates was performed at 20 x magnification for approximately 2 h with an interval of 15 s, using the IncuCyte Zoom imaging system (Sartorius, Göttingen, Germany).

### RNA isolation and RT-qPCR of MCPyV-positive and MCPyV-negative cell lines

Total cell lysates were obtained and RNA was extracted from a near confluent T25 flask for the adherent cell lines and approximately 3 × 10^6^ non-adherent cells from a T25 flask using TRIzol reagent (Invitrogen, Waltham, USA) according to the manufacturer’s instructions. The RNA pellets were dissolved in 50 µL nuclease-free water and the concentration was quantified using the Nanodrop spectrophotometer (Nanodrop 2000 C; Thermo Fisher Scientific, Waltham, USA). RNA samples were stored at -80 °C. 1 µg RNA of each cell line was converted into cDNA using the iScript cDNA Synthesis Kit (Bio-Rad Laboratories, Hercules, USA). The cDNA samples were stored in nuclease-free water at -20 °C.

The transcription levels for A- and B-type lamins in the cDNA samples were analyzed by qPCR using the specific primers as listed in Supplemental Table S4. Triplicate qPCRs were performed with all cDNA samples in triplicate. The qPCR was performed using the SensiMix SYBR Hi-ROX Kit (Meridian Bioscience, Cincinnati, USA) on a LightCycler480 (Roche Diagnostics, Basel, Switzerland) with the following program: activation 10 min at 95 °C, 40 cycles each of 15 s at 95 °C and 45 s at 60 °C, and a melting curve of 0.11 °C/sec from 65 °C to 95 °C. The Ct-values were calculated relative to the housekeeping gene hypoxanthine-guanine phosphoribosyltransferase (HPRT) with the formula 2^-ΔCt^. When the Ct-values were above 35 the lamin cDNA level was considered to be undetectable. For statistical analyses, two-tailed Student’s t-tests assuming unequal variances were performed. *P*-values ≤ 0.05 were considered statistically significant. Exact *p*-values are indicated in the text and indicated in the figures as follows: * for *p* ≤ 0.05, ** for *p* ≤ 0.01.

### RNA isolation and sequencing of MCC13 cells transfected with constructs encoding MCPyV-sT and LT antigens

RNA was isolated from 1 × 10^6^ transfected MCC13 cells, MKL-1, and MKL-2 using the Allprep DNA/RNA kit (Qiagen, Hilden, Germany). Sequencing and library preparation were handled by GenomeScan (GenomeScan BV, Leiden, The Netherlands), as described by Macamo et al.^78^. The sequencing output (fastq. gz files) was aligned to the human reference genome GRCh38 using the splice-aware aligner STAR (version 2.4.5b)^110^. The alignment was performed as a two-pass alignment according to the TCGA project. Transcript structure recovery and abundance estimation were carried out using Stringtie (version 2.2.0)^111^ and results were reported as transcripts per million (TPM).

### Western blotting

Total protein lysates were prepared using a near confluent T75 flask for the adherent cell lines and approximately 1 × 10^7^ non-adherent cells (also one T75 flask). Adhering cells were washed once with PBS and directly lysed on ice in radioimmunoprecipitation assay (RIPA) buffer containing 30 mM Tris-HCl, 50 mM NaCl, 1% Nonidet P 40 Substitute (NP40), 0.05% sodium deoxycholate, 0.1% sodium dodecyl sulfate (SDS), and protease inhibitors (Halt protease and phosphatase inhibitor single-use cocktail; Thermo Fisher Scientific). After 5 min cell remnants were scraped with a rubber policeman and the lysate was incubated on ice for 30 more min. Non-adherent cells were spun down, the pellet was washed once in PBS, resuspended in RIPA buffer, and incubated on ice for 30 min. The cell samples were diluted at a 1:1 ratio with sample buffer (62.5 mM Tris-HCl, 10% glycerol, 2.3% SDS, 5% β-mercaptoethanol, and 0.05% bromophenol blue), sonicated by adding glass beads (3–4 beads/sample, 2 mm size), and vortexing the mixture for 15 s. Before gel electrophoresis the samples were boiled at 95 °C for 10 min and centrifuged for 5 min at 12,000 g. Gel electrophoresis was performed in 8% SDS-polyacrylamide gels during 40 min at 200 V and the separated proteins were subsequently transferred onto nitrocellulose sheets (Protran; Schleicher & Schuell BioScience, Dassel, Germany) for 1 h at 100 V in blotting buffer (1× Tris-glycine buffer containing 20% methanol). After blotting, the nitrocellulose sheets were blocked using 5% non-fat dry milk solution (Difco Skim Milk; BD Life Sciences, New Jersey, USA). Protein detection was performed by overnight incubation with primary antibodies at 4 °C. Antibodies against lamins, emerin, and nuclear TATA binding protein TBP1 (loading control) were diluted in 2% non-fat dry milk solution as specified in Supplemental Table S5. After washing with Tris Buffered Saline with 0.2% Tween (TBST) (3 × 5 min), the blot was incubated with HRP-conjugated secondary antibodies, diluted in 2% non-fat dry milk solution as specified in Supplemental Table S6, on a shaking incubator for 1 h at RT. After washing with TBST (3 × 5 min) the enhanced chemiluminescence western blotting detection kit (SuperSignal West Pico Chemiluminescent Substrate; Thermo Fisher Scientific) and the Bio-Rad ChemiDoc XRS+ imaging system (Bio-Rad Laboratories) were used to detect the proteins of interest. The Precision Plus Protein Dual Color Standard (Bio-Rad Laboratories) was used as a molecular weight reference. Uncropped and unprocessed blots are shown in Supplemental Figure S4. Other exposure times not demonstrated in Fig. 2 are displayed in Supplemental Figures S5-S7.

### Sanger sequencing

MCC13 and MCC26 were analyzed using Sanger sequencing to investigate the presence of mutations or variants in lamin protein-encoding exons of the LMNA/C gene (NM_17070).

## Supplementary Information

Below is the link to the electronic supplementary material.

Supplementary Material 1

Supplementary Material 2

Supplementary Material 3

Supplementary Material 4

Supplementary Material 5

## Data Availability

The RNA sequencing datasets generated and/or analyzed during the current study are available in the Gene Expression Omnibus at https://www.ncbi.nlm.nih.gov/geo reference number GSE214948.
