# Supplementary material for: Variable A-type lamin expression in Merkel cell carcinoma cell lines and its association with nuclear integrity
Source: Sci Rep. 2026 Feb 25;16:11070. doi: 10.1038/s41598-026-39775-y (PMC13043713; doi:10.1038/s41598-026-39775-y)
Supplement: Supplementary file 5 — Supplementary Material 5 [file 41598_2026_39775_MOESM5_ESM.docx]

**Supplemental Data**

*Supplemental figures*


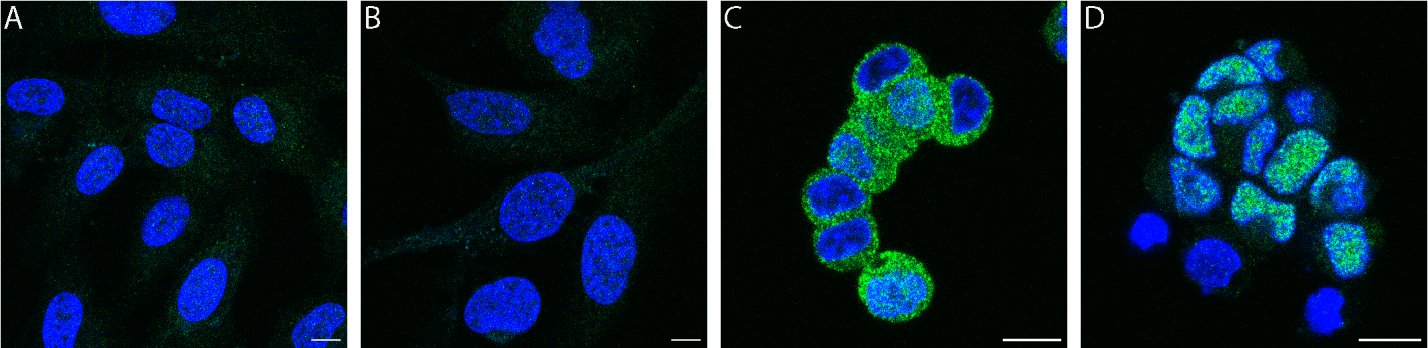
***Supplemental Figure S1:*** *Indirect immunofluorescence study showing that MCPyV-negative cell lines do not express MCPyV-LT, while MCPyV-positive cell lines MKL-1 and MKL-2 express MCPyV-LT.* ***A-D****) CLSM images of MCPyV-LT immunostaining (green), counterstained with DAPI (blue).* ***A****) MCC13;* ***B****) MCC26;* ***C****) MKL-1;* ***D****) MKL-2. Scale bars: 10 µm.*

***
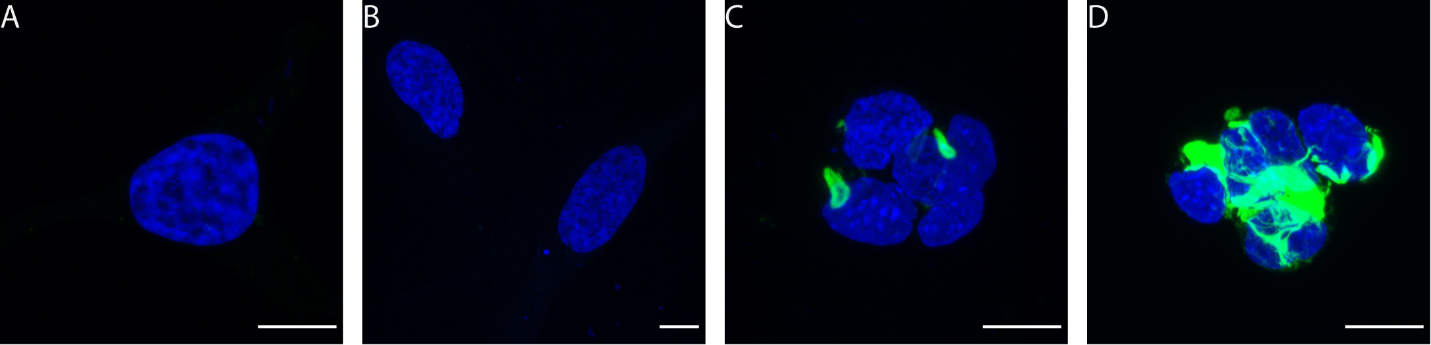
Supplemental Figure S2:*** *Indirect immunofluorescence study showing that MCPyV-negative cell lines MCC13 and MCC26 do not express CK20, while MCPyV-positive cell lines MKL-1 and MKL-2 express CK20.* ***A-D****) Maximal z-projections of CLSM images of CK20 immunostaining (green), counterstained with DAPI (blue).* ***A****) MCC13;* ***B****) MCC26;* ***C****) MKL-1;* ***D****) MKL-2. Scale bars: 10 µm.*

**
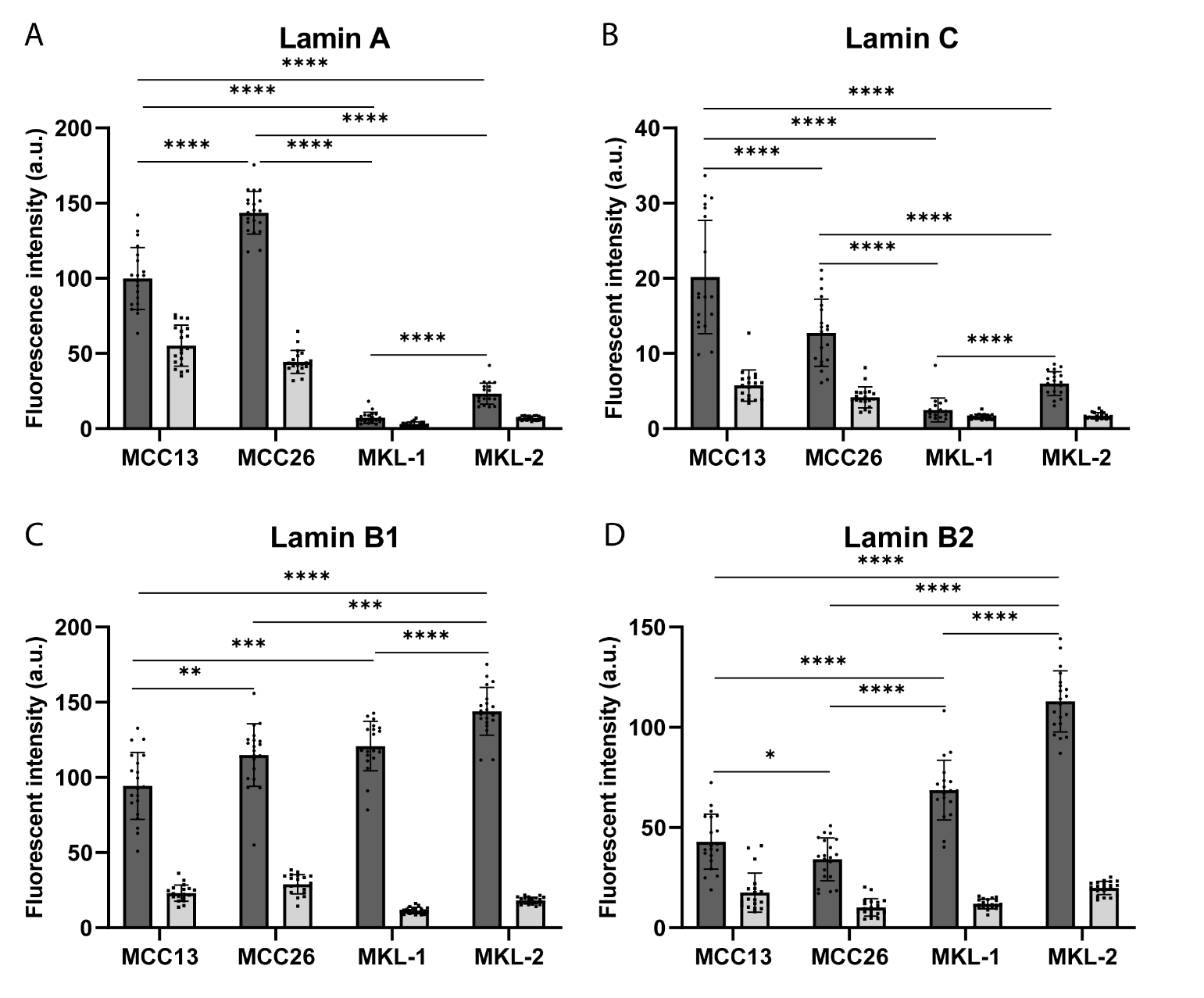
**

***Supplemental Figure S3:*** *Differential lamin expression in MCPyV-negative cell lines MCC13 and MCC26 as compared to MCPyV-positive cell lines MKL-1 and MKL-2, indicated as absolute fluorescence intensity values measured in the nuclear lamina and in the nucleoplasm. Maximum intensity values were measured using single z-slice CLSM images as shown in Figure 1.* ***A****) Lamin A;* ***B****) Lamin C;* ***C****) Lamin B1;* ***D****) Lamin B2. Data is represented as mean ± SD, n=20* *nuclei per cell line. *p ≤ 0.05, **p ≤ 0.01, ***p ≤ 0.001, **** p ≤ 0.0001. The significance levels between the different cell lines were as follows:*

*Lamin A: MCC13 vs. MCC26 p=4.6*10^-9^, MCC13 vs. MKL-1 p=1.1*10^-14^, MCC13 vs. MKL-2 p=6.2*10^-14^, MCC26 vs. MKL-1 p=4.4*10^-22^, MCC26 vs. MKL-2 p=2.9*10^-24^, MKL-1 vs. MKL-2 p=7.6*10^-10^.*

*Lamin C: MCC13 vs. MCC26 p=6.4*10^-4^, MCC13 vs. MKL-1 p=1.4*10^-9^, MCC13 vs. MKL-2 p=6.0*10^-8^, MCC26 vs. MKL-1 p=1.0*10^-9^, MCC26 vs. MKL-2 p=1.5*10^-6^, MKL-1 vs. MKL-2 p=2.4*10^-8^.*

*Lamin B1: MCC13 vs. MCC26 p=4.6*10^-3^, MCC13 vs. MKL-1 p=1.4*10^-4^, MCC13 vs. MKL-2 p=1.8*10^-9^, MCC26 vs. MKL-2 p=1.8*10^-5^, MKL-1 vs. MKL-2 p=5.7*10^-5^.*

*Lamin B2: MCC13 vs. MCC26 p=2.9*10^-2^, MCC13 vs. MKL-1 p=1.5*10^-6^, MCC13 vs. MKL-2 p=1.0*10^-17^, MCC26 vs. MKL-1 p=6.7*10^-10^, MCC26 vs. MKL-2 p=1.1*10^-19^, MKL-1 vs. MKL-2 p=2.6*10^-11^.*

***
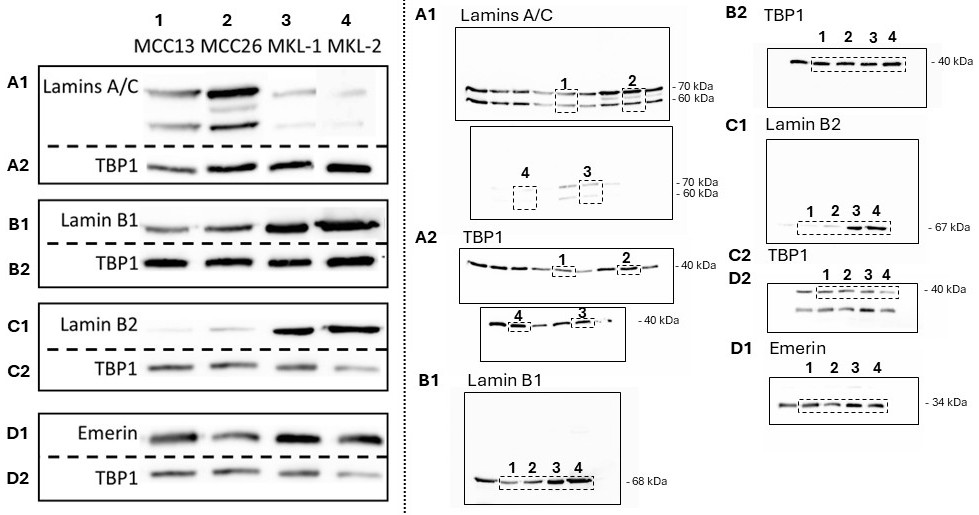
***

***Supplemental Figure S4:*** *Unprocessed and uncropped blots of Figure 2. Left part of the image shows the original Figure 2. Right part of the image displays the original immunoblots used to produce Figure 2. Black lines visualize the borders of the (cut) blot.* ***A1****) lamins A/C.* ***B1****) lamin B1.* ***C1****) lamin B2.* ***D1****) Emerin.* ***A2, B2, C2, D2****) TBP1 was used as loading control, which has been performed on the same blots as the proteins of interest, but detection was performed on separated blot parts. The different samples tested for lamins A/C (A1) and the loading control TBP1 (****A2****) were divided over two blots, but stained and detected simultaneously. Lamin B2, emerin and TPB1 (****C1, C2, D1, D2****) were detected on the same blot, with TBP1 antibody incubation and detection (****C2, D2****) being performed after emerin.*

*
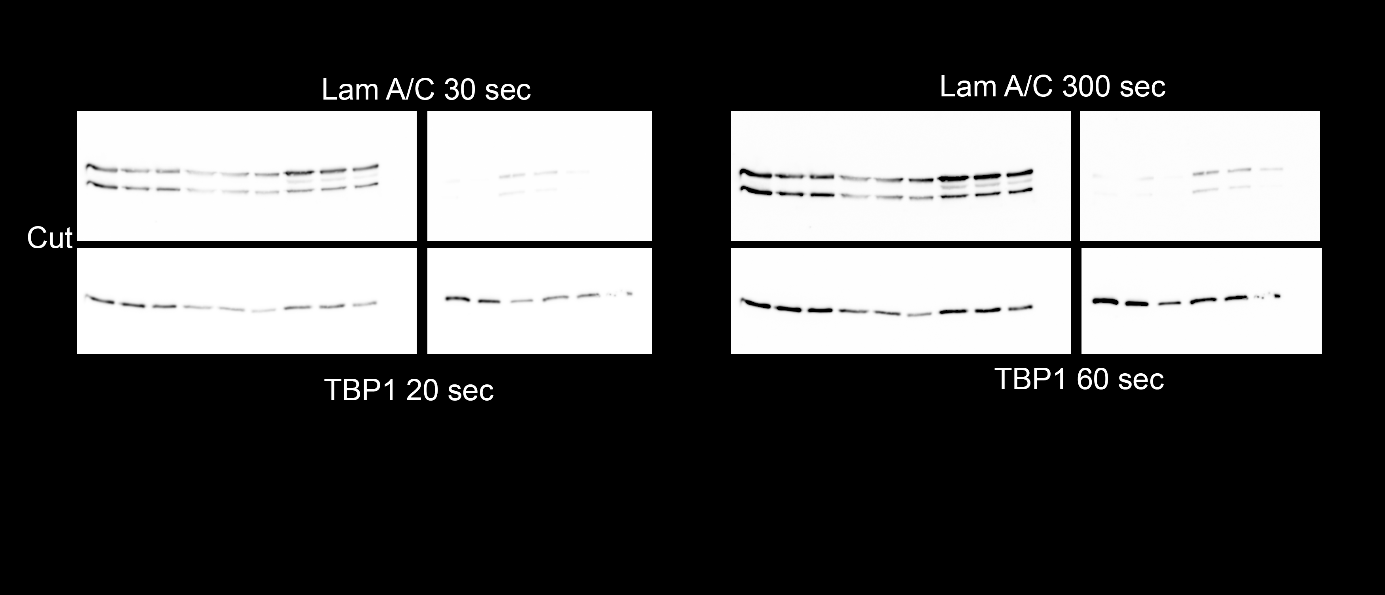
*

***Supplemental Figure S5:*** *Additional exposure times for Western blot of lamins A/C and the loading control TBP1 (exposure time in Figure 2 and Supplemental Figure S4 of lamins A/C : 1330 sec, TBP1: 60 sec). The different samples tested for lamins A/C (A1) and the loading control TBP1 (****A2****) were divided over two blots, but stained and detected simultaneously.*

*
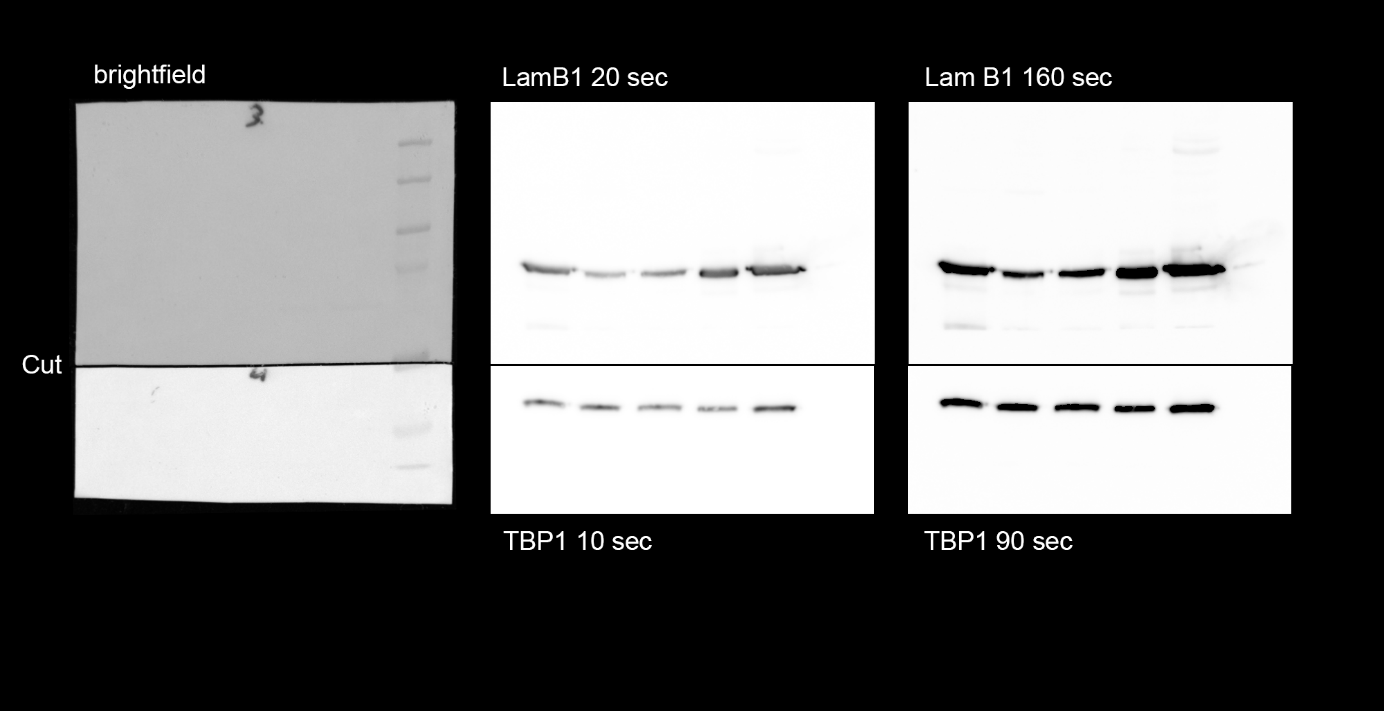
*

***Supplemental Figure S6:*** *Additional exposure times for Western blot of lamin B1 and the loading control TBP1 (exposure time in Figure 2 and Supplemental Figure S4 of lamin B1: 80 sec, TBP1: 90 sec). Brightfield image on the left displays that lamin B1 and TBP1 are detected on the same original blot, but the blot was cut before antibody incubation.*

*
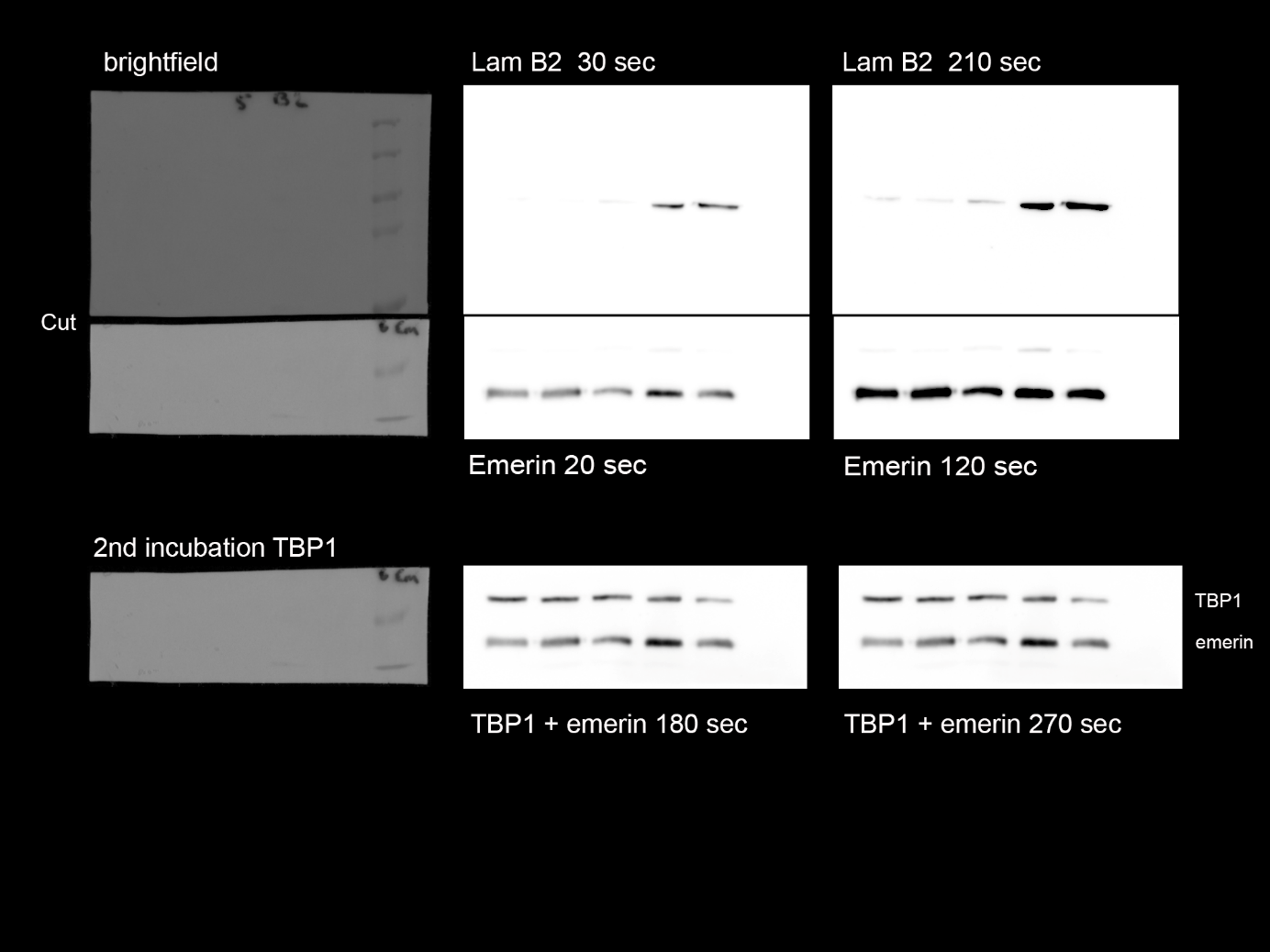
*

***Supplemental Figure S7:*** *Additional exposure times for Western blot of lamin B2, emerin and the loading control TBP1 (exposure time in Figure 2 and Supplemental Figure S4 of lamin B2: 210 sec, emerin: 60 sec, TBP1: 180 sec). Brightfield image on the left displays that lamin B2, emerin and TBP1 are detected on the same original blot, but the blot was cut before antibody incubation. Antibody incubation and detection was first performed for emerin, followed for TBP1.*


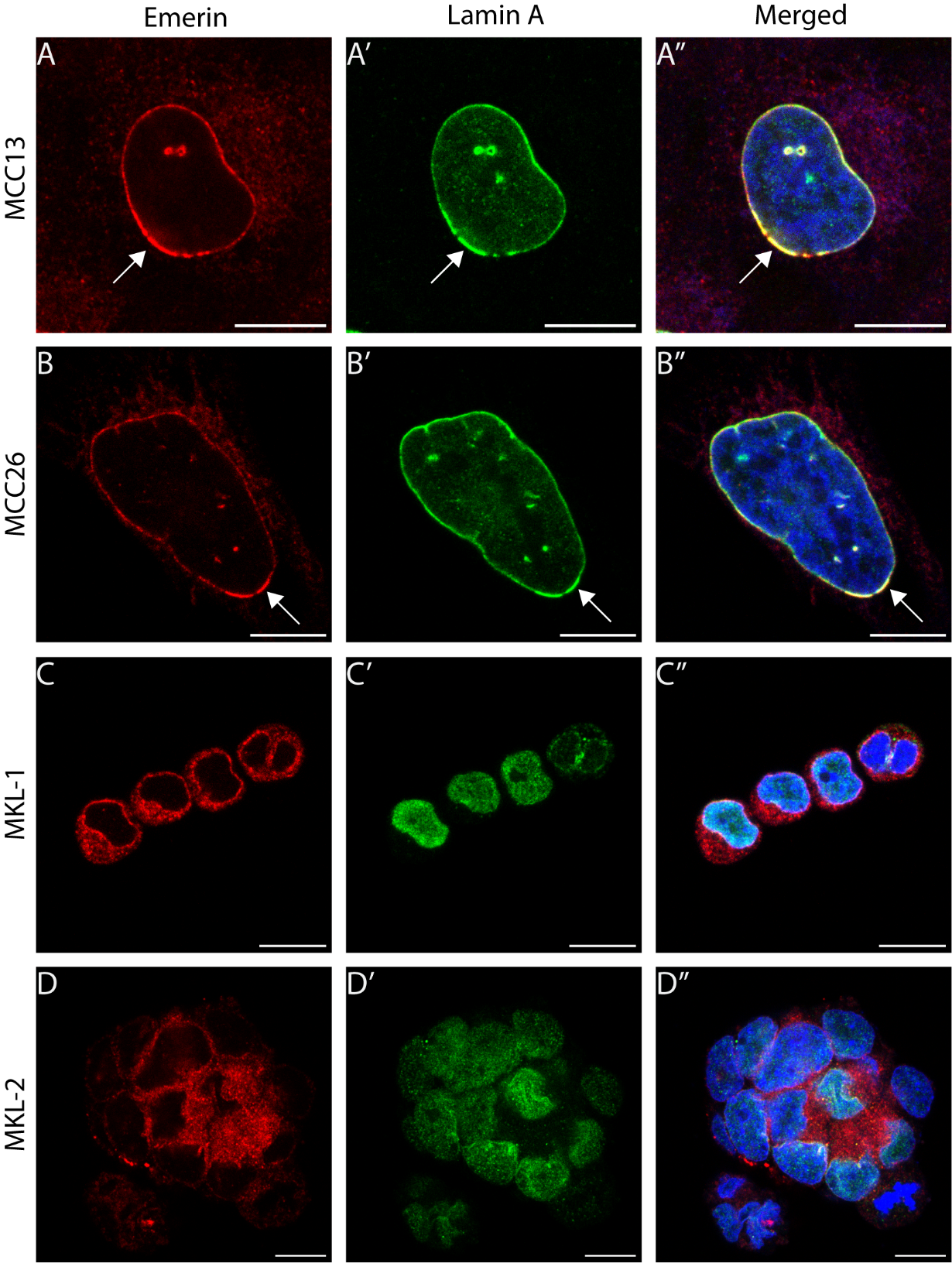


***Supplemental Figure S8:*** *Local accumulation of lamin A and re-localization of emerin in MCC cell lines. CLSM images of emerin (****A-D****) and lamin A (****A’-D’****) immunostaining in MCC13 (****A****), MCC26 (****B****), MKL-1 (****C****), and MKL-2 (****D****). Emerin labelling demonstrates the main localization at the NE in MCPyV-negative cell lines, but with local accumulations (see arrows in* ***A, B****), while cytoplasmic localization, most likely representing emerin immunostaining in the endoplasmic reticulum, is seen in the MCPyV-positive cell lines (****C, D****). Lamin A labelling also demonstrates local accumulations (see arrows in* ***A’, B’****), which overlap with the emerin accumulations, as visible in the merged images (****A’’-B’’****). Merged images (****A’’-D’’****) visualize emerin (red), lamin A (green) and DAPI (blue). The brightness of the images was enhanced to illustrate the differences in lamin and emerin distribution. Scale bars: 10 μm.*


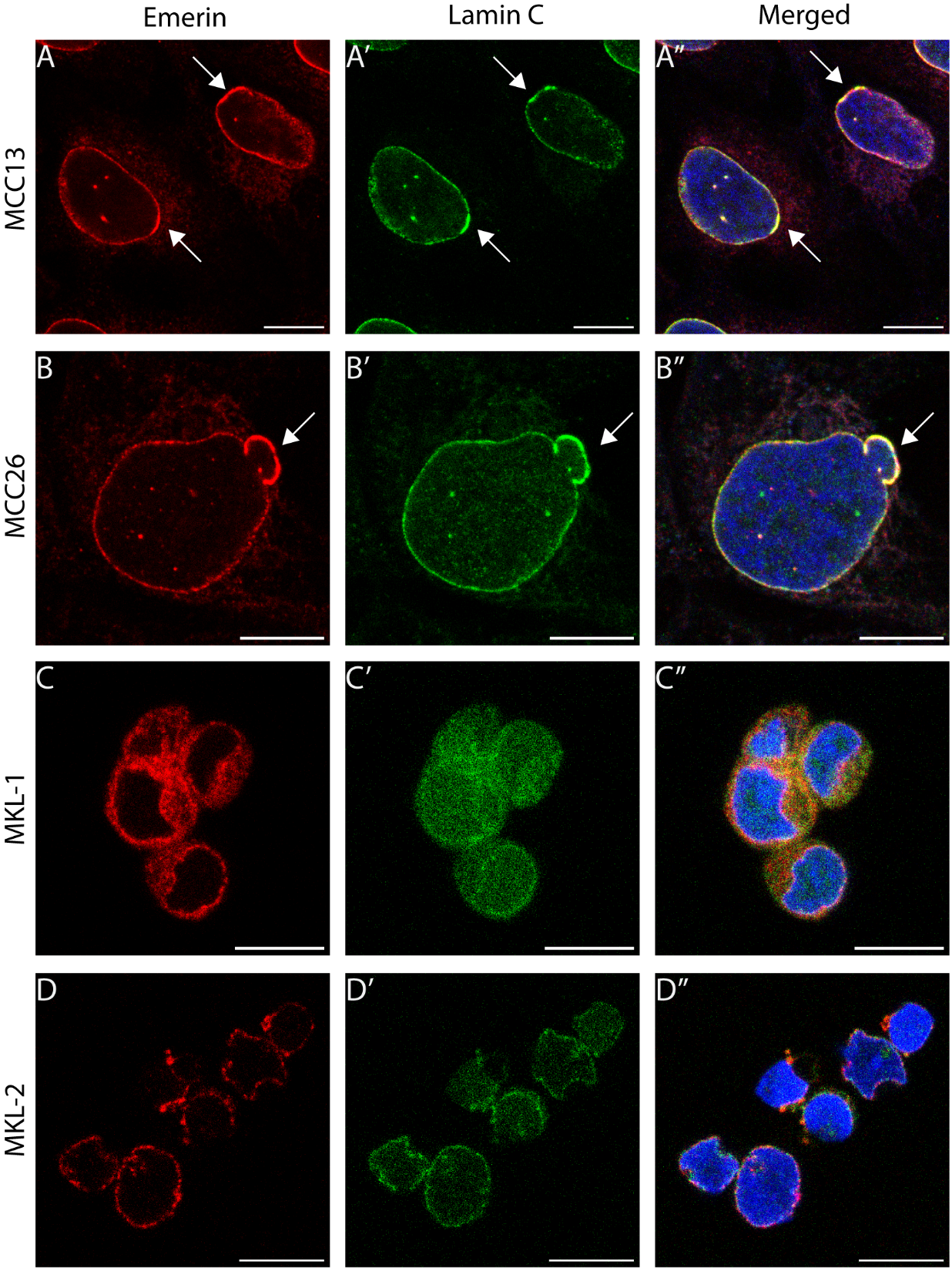


***Supplemental Figure S9:*** *Local accumulation of lamin C and re-localization of emerin in MCC cell lines. CLSM images of emerin (****A-D****) and lamin C (****A’-D’****) immunostaining in MCC13 (****A****), MCC26 (****B****), MKL-1 (****C****), and MKL-2 (****D****). Emerin labelling demonstrates the main localization at the NE in MCPyV-negative cell lines, but with local accumulations (see arrows in* ***A, B****), while cytoplasmic localization, most likely representing emerin immunostaining in the endoplasmic reticulum, is seen in the MCPyV-positive cell lines (****C, D****). Lamin C labelling also demonstrates local accumulations (see arrows in* ***A’, B’****), which overlaps with the emerin accumulations, as visible in the merged images (****A’’-B’’****). Merged images (****A’’-D’’****) visualize emerin (red), lamin C (green) and DAPI (blue). The brightness of the images was enhanced to illustrate the differences in lamin and emerin distribution. Scale bars: 10 μm.*

*
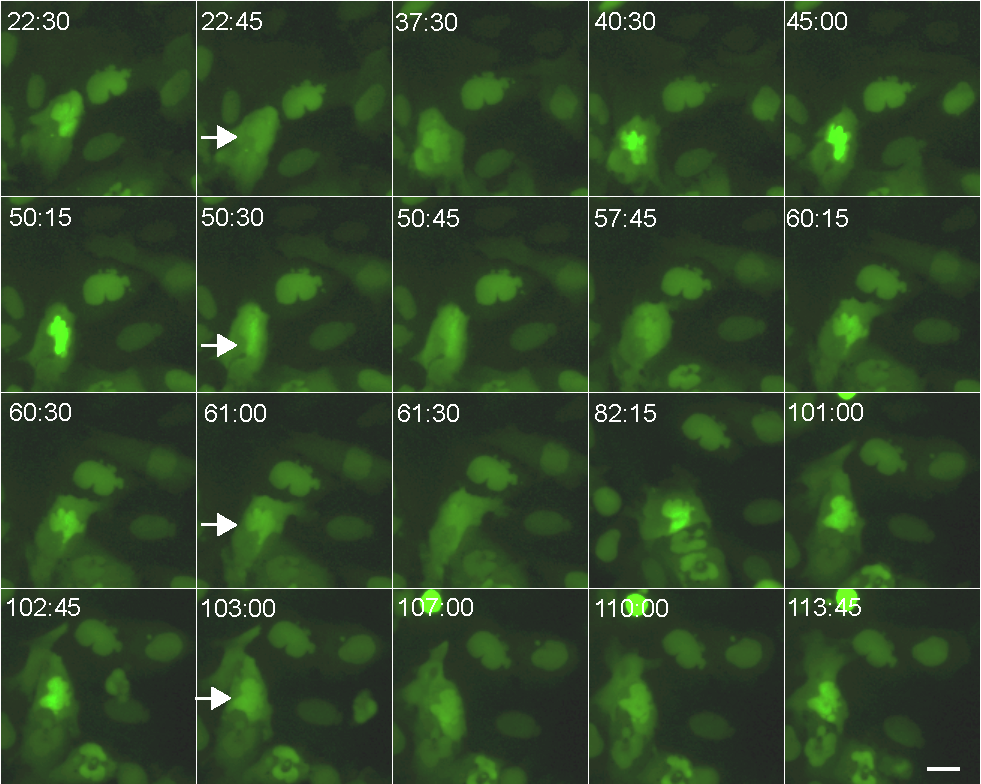
*

***Supplemental Figure S10:*** *Repetitive nuclear envelope (NE) ruptures in MCC26.* ***A****) Selected images from a recording (Supplemental Video S2) of an MCC26 cell transfected with the construct encoding NLS-EYFP, using the IncuCyte Zoom imaging system. The second column from the left (see arrows) displays NE rupture events, (partial) restoration of the nuclear signal is visible in the following columns of the same row. Note the different time windows in which restoration of the nuclear signal takes place. Time passaged in minutes since initiation of recording is indicated at the top left corner. Scale bar represents 10 μm.*


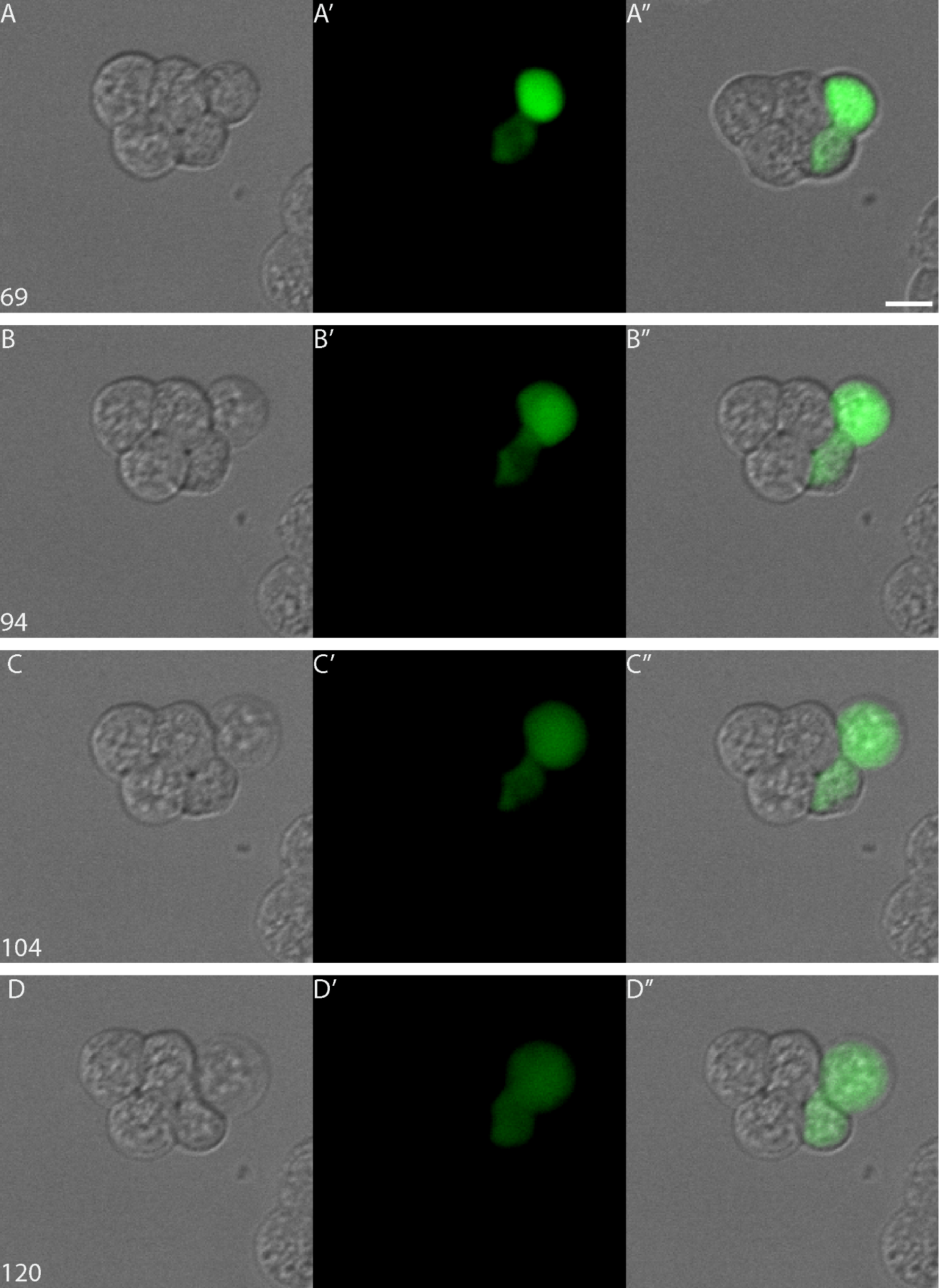


***Supplemental Figure S11:*** *Nuclear envelope (NE) rupture in MKL-1. Selected images from a recording (Supplemental Video S4) of MKL-1 cells transfected with the construct encoding NLS-EYFP, using the vital imaging system. Differential Interference Contrast (DIC) images (****A-D****), fluorescence images (****A’-D’****), and merged images (****A’’-D’’****) demonstrate that an NE rupture occurs at 94 minutes after initiation of recording. Frame rate: one image per minute, 120 frames in total. Scale bar: 10 µm.*


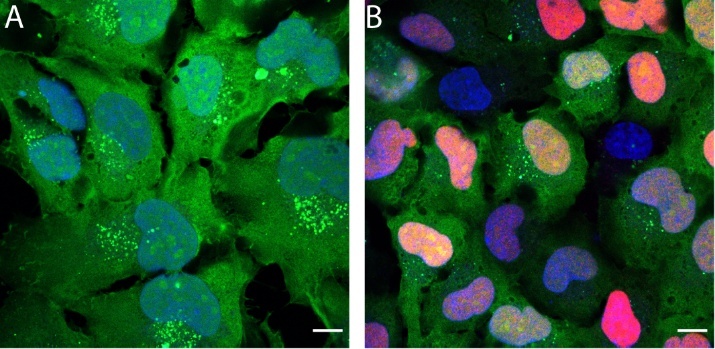


***Supplemental Figure S12:*** *MCPyV-negative cell line MCC13 transfected with the construct encoding MCPyV-LT is positive for LT, while MCC13 transfected with empty vector (EV) is negative. CLSM images of MCC13EV (****A****) and MCC13LT (****B****) stained for LT (red) and counterstained with DAPI (blue). The cytoplasmic green staining of the cells indicates that the cells express green fluorescent protein (GFP) that is present in all constructs as a positive transfection control. Scale bars: 10 µm.*


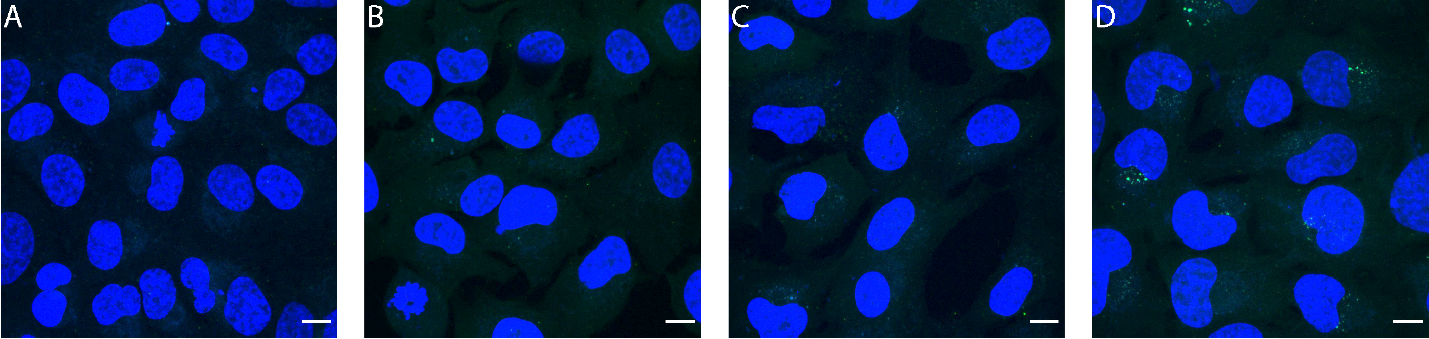


***Supplemental Figure S13:*** *MCPyV-negative cell line MCC13 transfected with the construct encoding MCPyV-LT, MCPyV-sT, or an empty vector (EV) are negative for CK20.* ***A-D****) Maximal z-projections of CLSM images of CK20 immunostaining (green), counterstained with DAPI (blue).* ***A****) MCC13;* ***B****) MCC13sT;* ***C****) MCC13LT;* ***D****) MCC13EV. Scale bars: 10 µm.*


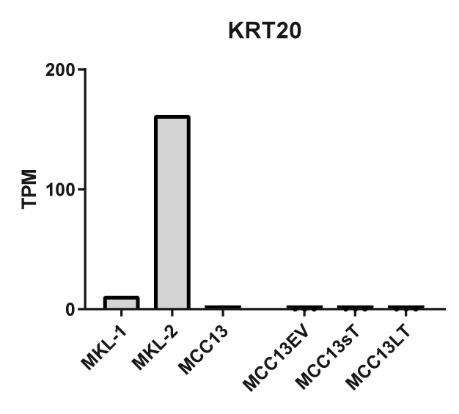


***Supplemental Figure S14:*** *Expression level (transcripts per million; TPM) of cytokeratin 20 (KRT20) based on RNA sequencing of MCPyV-positive cell lines MKL-1 and MKL-2 and in MCPyV-negative cell line MCC13, either non-transfected or transfected with the construct encoding MCPyV-LT (MCC13LT), MCPyV-sT (MCC13sT) or an empty vector (MCC13EV). MCC13LT, MCC13sT, and MCC13EV were analyzed in triplicate, data represents mean ± SD.*


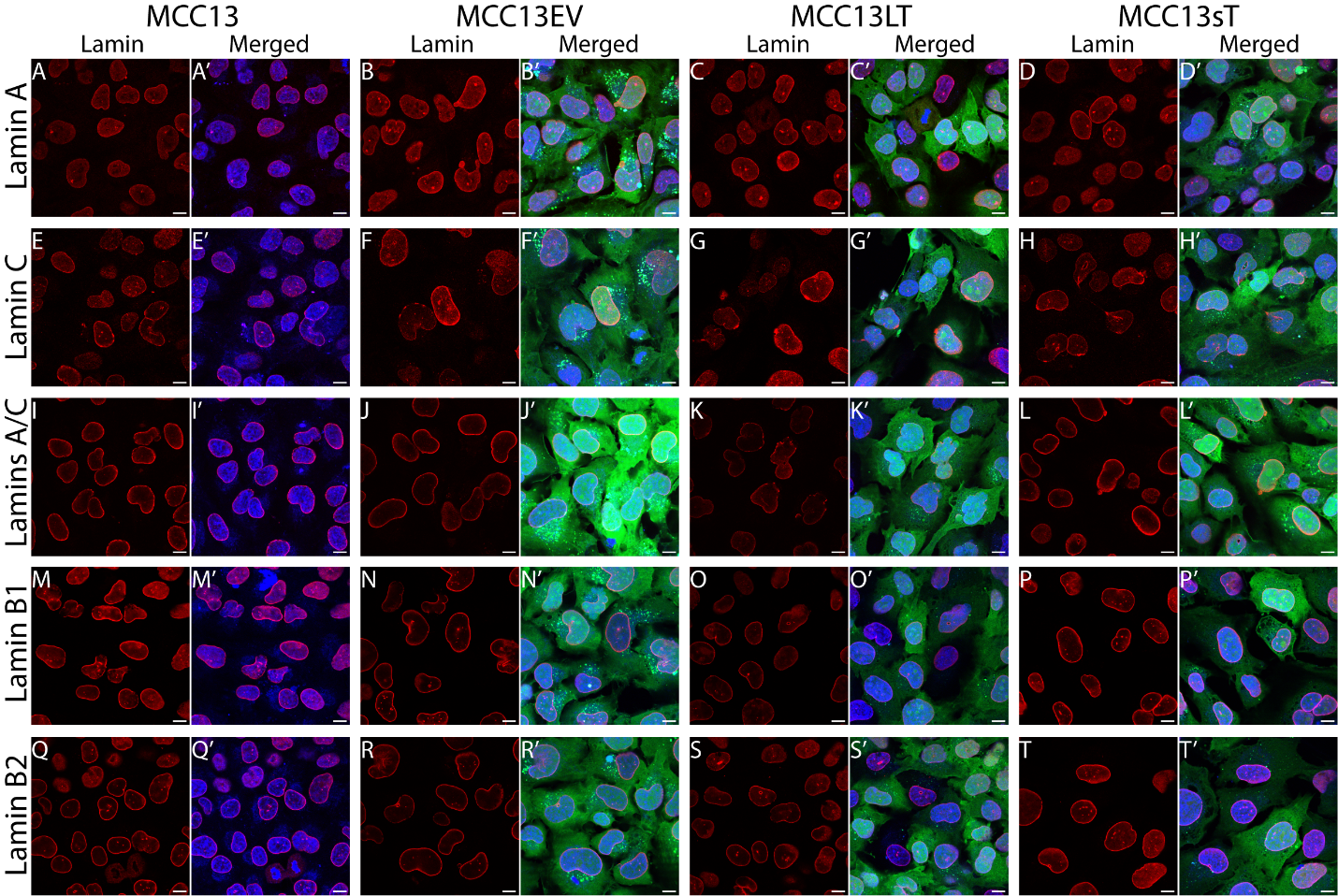


***Supplemental Figure S15:*** *Lamin expression in MCPyV-negative cell line MCC13 transfected with the constructs encoding MCPyV-LT, MCPyV-sT or an empty vector (EV).* ***A-T****) CLSM images of lamin A (****A-D****), lamin C (****E-H****), lamins A/C (****I-L****), lamin B1 (****M-P****), and lamin B2 (****Q-T****) immunostaining in MCC13 (****A, E, I, M, Q****), MCC13EV (****B, F, J, N, R****), MCC13LT (****C, G, K, O, S****), and MCC13sT (****D, H, L, P, T****).* ***A’-T’****) CLSM images of (****A-T****) (red) merged with DAPI counterstain of nuclei (blue) and green fluorescent protein signal (green), which indicates successful transfection. For each antibody staining the fluorescence intensities of lamin are scaled to the same value. Scale bars 10 µm.*

*Supplemental videos*

***Supplemental Video S1:*** *Repetitive nuclear envelope ruptures and restoration of nuclear signal in MCC13 cells transfected with a construct encoding NLS-EYFP. Frame rate: one image per 15 seconds, 494 frames in total. Selected images are shown in Figure 7.*

***Supplemental Video S2:*** *Repetitive nuclear envelope ruptures and restoration of nuclear signal in MCC26 cells transfected with a construct encoding NLS-EYFP. Frame rate: one image per 15 seconds, 494 frames in total. Selected images are shown in Supplemental Figure S6.*

***Supplemental Video S3:*** *Nuclear envelope rupture and cell death in MKL-2 cells transfected with a construct encoding NLS-EYFP. Video displays, from left to right: Differential Interference Contrast (DIC) images, fluorescence images, and merged images. Frame rate: one image per minute, 58 frames in total. NE rupture occurs at 28 minutes after initiation of the recording. Selected images are shown in Figure 8.*

***Supplemental Video S4:*** *Video of nuclear envelope rupture and cell death in MKL-1 cells transfected with a construct encoding NLS-EYFP. Video displays merged images of Differential Interference Contrast (DIC) images and fluorescence images at a frame rate of one image per minute, 120 frames in total. NE rupture occurs at 94 minutes after initiation of recording. Selected images are shown in Supplemental Figure S7.*

*Supplemental tables*

***Supplemental Table S1:*** *Ratio between nuclear lamina and nucleoplasmic lamin signal in MCPyV-negative and MCPyV-positive cells as determined by quantification of CLSM images of lamin immunostainings (Figure 1 and Supplemental Figure S3).*

| **Lamin type** | **MCC13** | **MCC26** | **MKL-1** | **MKL-2** |
| --- | --- | --- | --- | --- |
| **Lamin A** | 1.9 ± 0.5 | 3.3 ± 0.5 | 2.4 ± 0.8 | 3.3 ± 0.9 |
| **Lamin C** | 3.6 ± 0.9 | 3.1 ± 1 | 1.5 ± 0.7 | 3.5 ± 0.7 |
| **Lamin B1** | 4.2 ± 0.8 | 4.1 ± 0.7 | 11 ± 2 | 8.0 ± 1 |
| **Lamin B2** | 2.7 ± 0.9 | 3.6 ± 1 | 5.9 ± 1 | 5.8 ± 1 |

***Supplemental Table S2:*** *Characteristics of primary antibodies used for immunofluorescence (IF) staining.*

| **Antibody** | **Antigen** | **Species, subclass** | **Dilution** | **Fixation** | **Provider** | **Cat. No.** |
| --- | --- | --- | --- | --- | --- | --- |
| **133A2** | Lamin A | Mouse monoclonal IgG3 | 1:2000 | 4% formaldehyde | Nordic-MUbio, Susteren, The Netherlands | MUB1101P |
| **Jol2** | Lamins A/C | Mouse monoclonal IgG1 | 1:50 | 4% formaldehyde | Gift from Prof. dr. C. Hutchinson, Durham, UK | - |
| **Lamin B1** | Lamin B1 | Rabbit polyclonal IgG | 1:1000 | Methanol | Abcam, Cambridge, UK | ab16048 |
| **LN43** | Lamin B2 | Mouse monoclonal IgG1 | Undiluted  culture sup. | Methanol | Gift from Prof. dr. E.B. Lane, Dundee, UK | - |
| **8F6-E8-F12** | Lamin B2 | Mouse monoclonal IgG1,к | 1:1000 | 4% formaldehyde | Biolegend, San Diego, USA | 871502 |
| **RalC** | Lamin C | Rabbit polyclonal IgG | 1:50 | Methanol/ 4% formaldehyde | Immuquest, Stokesley, UK | IQ177 |
| **4G5** | Emerin | Mouse monoclonal IgG1 | 1:50 | 4% formaldehyde | Novocastra, Newcastle upon Tyne, UK | NCL-EMERIN |
| **PG-M3** | PML Nuclear Bodies | Mouse monoclonal IgG1 | 1:100 | 4% formaldehyde | Santa Cruz Biotechnology, Heidelberg, Germany | sc-966 |
| **IT-Ks20.8** | Cytokeratin 20 | Mouse monoclonal IgG2a | 1:100 | 4% formaldehyde | Progen, Heidelberg, Germany | 690026 |
| **CM2B4** | Large T antigen | Mouse recombin.  monoclonal | 1:1000 | 4% formaldehyde | Abcam, Cambridge, UK | ab307450 |

***Supplemental Table S3:*** *Characteristics of secondary antibodies used for immunofluorescence (IF) staining.*

| **Antibody** | **Dilution** | **Provider** | **Cat. No.** |
| --- | --- | --- | --- |
| Swine anti-rabbit Ig-FITC | 1:100 | Dako/Agilent, Glostrup, Denmark | F0205 |
| Rabbit anti-mouse Ig-FITC | 1:100 | Dako/Agilent, Glostrup, Denmark | F026102 |
| Goat anti-mouse IgG3-FITC | 1:50 | SouthernBiotech, Birmingham, USA | 1100-02 |
| Goat anti-mouse IgG-Alexa Fluor 568 | 1:500 | Life Technologies, Eugene, USA | A11031 |
| Goat anti-rabbit IgG-Alexa Fluor 568 | 1:500 | Life Technologies, Eugene, USA | A11036 |
| Goat anti-mouse IgG1-Texas Red | 1:50 | SouthernBiotech, Birmingham, USA | 1070-07 |

***Supplemental Table S4:*** *Primer sequences of the qPCR primer pairs applied in this study.*

| **Protein (gene)** | **Primer sequence** |
| --- | --- |
| **Lamin A (*LMNA*)** | Forward primer: 5’- TCTTCTGCCTCCAGTGTCACG-3’  Reverse primer: 5’-AGTTCTGGGGGCTCTGGGT-3’ |
| **Lamin C (*LMNA*)** | Forward primer: 5’-CAACTCCACTGGGGAAGAAGTG-3’  Reverse primer: 5’-CGGCGGCTACCACTCAC-3’ |
| **Lamin B1 (*LMNB1*)** | Forward primer: 5'-AAGGCGAAGAAGAGAGGTTGAAG-3'  Reverse primer: 5'-GCGGAATGAGAGATGCTAACACT-3' |
| **Lamin B2 (*LMNB2*)** | Forward primer: 5’-TGGAGATCAACGCCTACCG-3’  Reverse primer: 5’-AGCCGCTTCCGCTTACTG -3’ |
| **HPRT (*HPRT*)** | Forward primer: 5’-CACTGGCAAAACAATGCAGACT-3’  Reverse primer: 5’-GTCTGGCTTATATCCAACACTTCGT-3’ |

***Supplemental Table S5:*** *Characteristics of primary antibodies used for Western blotting.*

| **Antibody** | **Antigen** | **Species, subclass** | **Dilution** | **Provider** | **Cat. No.** |
| --- | --- | --- | --- | --- | --- |
| **133A2** | Lamin A | Mouse monoclonal IgG3 | 1:5000 | Nordic-MUbio, Susteren, The Netherlands | MUB1101P |
| **Jol2** | Lamins A/C | Mouse monoclonal IgG1 | 1:100 | Gift from Prof. dr. C. Hutchinson, Durham, UK | - |
| **Lamin B1** | Lamin B1 | Rabbit polycloxnal IgG | 1:15000 | Abcam, Cambridge, UK | ab16048 |
| **LN43** | Lamin B2 | Mouse monoclonal IgG1 | 1:200 | Gift from Prof. dr. E.B. Lane, Dundee, UK | - |
| **4G5** | Emerin | Mouse monoclonal IgG1 | 1:1000 | Novocastra, Newcastle upon Tyne, UK | NCL-EMERIN |
| **1TBP18** | TATA binding protein TBP1 | Mouse monoclonal IgG1 | 1:5000 | Abcam, Cambridge, UK | ab818 |

***Supplemental Table S6:*** *Characteristics of secondary antibodies used for Western blotting.*

| **Antibody** | **Dilution** | **Provider** | **Cat. No.** |
| --- | --- | --- | --- |
| Goat anti-mouse IgG-HRP | 1:3000 | Cell Signalling Technology Europe, Leiden, The Netherlands | 7076 |
| Goat anti-rabbit IgG-HRP | 1:3000 | Cell Signalling Technology Europe, Leiden, The Netherlands | 7074 |
